# A novel *N*^6^-Deoxyadenine methyltransferase METL-9 modulates *C. elegans* immunity via dichotomous mechanisms

**DOI:** 10.1038/s41422-023-00826-y

**Published:** 2023-06-05

**Authors:** Chengchuan Ma, Tingling Xue, Qi Peng, Jie Zhang, Jialiang Guan, Wanqiu Ding, Yi Li, Peixue Xia, Liankui Zhou, Tianyu Zhao, Sheng Wang, Li Quan, Chuan-Yun Li, Ying Liu

**Affiliations:** 1 State Key Laboratory of Membrane Biology, New Cornerstone Science Laboratory, Institute of Molecular Medicine, College of Future Technology, Peking University, Beijing, China; 2 Peking-Tsinghua Center for Life Sciences, Peking University, Beijing, China; 3 Beijing Advanced Innovation Center for Genomics, Beijing, China; 4 Research Center for Stem Cell and Regenerative Medicine, Institute of Blood Transfusion, Chinese Academy of Medical Sciences and Peking Union Medical College, Chengdu, Sichuan China; 5 Laboratory of Bioinformatics and Genomic Medicine, Institute of Molecular Medicine, College of Future Technology, Peking University, Beijing, China; 6 PKU-Tsinghua-NIBS Graduate Program, Academy for Advanced Interdisciplinary Studies, Peking University, Beijing, China; 7 Shanghai Zelixir Biotech Company Ltd., Shanghai, China

**Keywords:** DNA methylation, Methylation analysis

## Abstract

*N*^6^-Methyldeoxyadenine (6mA) has been rediscovered as a DNA modification with potential biological function in metazoans. However, the physiological function and regulatory mechanisms regarding the establishment, maintenance and removal of 6mA in eukaryotes are still poorly understood. Here we show that genomic 6mA levels change in response to pathogenic infection in *Caenorhabditis elegans* (*C. elegans*). We further identify METL-9 as the methyltransferase that catalyzes DNA 6mA modifications upon pathogen infection. Deficiency of METL-9 impairs the induction of innate immune response genes and renders the animals more susceptible to pathogen infection. Interestingly, METL-9 functions through both 6mA-dependent and -independent mechanisms to transcriptionally regulate innate immunity. Our findings reveal that 6mA is a functional DNA modification in immunomodulation in *C. elegans*.

## Introduction

The ability to sense pathogen infection and initiate immune responses is crucial for organismal survival. Eukaryotes have evolved multiple and complex mechanisms for immunomodulation to maintain organismal homeostasis.^1,2^

Epigenetic modifications such as histone acetylation, histone methylation and DNA methylation have been reported to play important roles in immune responses across the phyla.^3,4^ Epigenetic modification can rapidly alter global gene expression levels, without the need to change genomic sequences, thereby allowing organisms to respond expeditiously and reversibly to the infection. DNA methylation is an evolutionarily conserved epigenetic modification that plays important roles in regulating gene expression. Compared with 5-methyldeoxycytosine (5mC) and its oxidation derivatives, which are considered as the predominant form of DNA methylation in higher eukaryotes, *N*^*6*^-methyldeoxyadenine (6mA) has only been rediscovered recently to be present and functional in eukaryotic genomes.^5–9^ However, recent literature casts doubts on the existence and physiological function of 6mA in eukaryotes. Concerns have centered on bacterial contamination, antibody cross-reactivity, and random incorporation of methyldeoxyadenine without meaningful function.^10–17^ Identification of 6mA modifiers and exploration of the regulatory mechanisms of 6mA are crucial to understand its physiological functions. Moreover, genetic approaches such as comparing 6mA levels and associated phenotypes in wild-type (WT) animals and methyltransferase mutant animals are critical to demonstrate the existence and physiological function of 6mA in eukaryotes. Previously, 6mA was reported to be associated with regulation of mitochondrial stress response and heat shock response in *Caenorhabditis elegans* (*C. elegans*),^18,19^ which suggests that 6mA may play an important role in modulating stress responses.

Here we show that the genomic 6mA level in *C. elegans* is upregulated upon pathogen infection. We further identify METL-9 as the 6mA methyltransferase responsible for pathogen infection-induced 6mA elevation. Knockout (KO) of METL-9 or generation of 6mA-catalytic dead METL-9 impairs 6mA induction following infection and results in defective immunity. METL-9 and 6mA transcriptionally regulate worm immunity. Transcript levels of immune response genes are dysregulated in METL-9 mutant animals. Collaborating with Zhang et al., we have developed a single-nucleotide-resolution single-molecule real-time sequencing (SMRT-seq) method, 6mA-Sniper, which reveals the genomic 6mA profile upon infection.^20^ Genes critical for immune response are modified and transcriptionally regulated by 6mA. Interestingly, animals expressing 6mA-catalytically dead METL-9 display residual immunity upon pathogen infection, which suggests that a methyltransferase activity-independent function of METL-9 is partially responsible for immunomodulation. Further exploration uncovered that METL-9 associates with the chromatin organizer protein DVE-1 to control the transcription of immune response genes. Our study generates clear evidence that 6mA is a functional epigenetic modification, which modulates gene transcription and controls innate immunity in *C. elegans*.

## Results

### Genomic 6mA level in *C. elegans* is increased upon infection

**Fig. 1:**
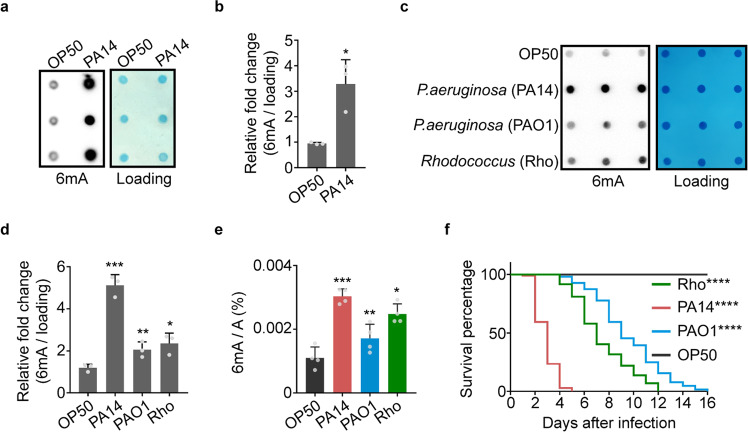
The level of genomic 6mA in *C. elegan*s is increased by pathogen infection. **a**–**d** 6mA dot blotting (**a**, **c**) and quantification (**b**, **d**) of WT animals under each indicated treatment. *n* = 3. Bars indicate means + SD. Two-tailed *t*-test, *P < 0.05, **P < 0.01, ***P < 0.001. **e** LC-MS/MS analysis of gDNA 6mA level in WT animals fed on OP50, PA14, PAO1 or *Rhodococcus*. *n* = 4. Bars indicate means + SD. Two-tailed *t*-test, *P < 0.05, **P < 0.01, ***P < 0.001. **f** Slow-killing assay of WT animals fed on OP50, PA14, PAO1 or *Rhodococcus*. *n* = 100. Log-rank (Mantel–Cox) test, ****P < 0.0001.

We previously reported that levels of 6mA change in response to mitochondrial stress, and 6mA participates in transgenerational inheritance of stress adaptation in *C. elegans*.^18^ Several other studies also reported dynamic changes of DNA 6mA modification under environmental stresses.^19,21,22^ We hypothesized that 6mA levels might respond to severe stresses and play critical roles in modulating stress responses. Immune responses are types of stress responses that are essential for organismal survival. Inability to activate immune responses has been implicated in many diseases. Therefore, we sought to test whether 6mA plays a role in initiating immune responses, and whether the 6mA levels in the genomic DNA (gDNA) of *C. elegans* respond to pathogenic infection. Interestingly, the gDNA 6mA levels were dramatically increased when animals were challenged with *Pseudomonas aeruginosa* strains (PA14 and PAO1) and a *Rhodococcus* strain (GRm0270) isolated from the natural habitat of *C. elegans*^23^ (Fig. 1a–e). Moreover, the fold changes of the 6mA levels strongly correlated with the severity of infection (Fig. 1c–f). The animals fed with PA14 had the shortest survival time and the greatest induction of gDNA 6mA, whereas the animals fed with PAO1 had the longest survival time and the least induction of gDNA 6mA. In addition, worms fed with *ΔgacA* PA14, a less pathogenic PA14 strain, revealed a remarkable reduction of 6mA induction compared with worms fed the WT PA14 (Supplementary information, Fig. S1a). These results suggest that the elevation of 6mA might be part of the innate immune response. Signaling by the PMK-1 p38 mitogen-activated protein kinase (MAPK) is one of the important pathways that modulate *C. elegans* innate immunity.^24,25^ Interestingly, the induction of genomic 6mA upon PA14 infection is comparable in *pmk-1* mutants to that in WT animals. This suggests that a *pmk-1*-independent mechanism is responsible for pathogen sensing and subsequent establishment of 6mA upon infection (Supplementary information, Fig. S1b). We also tested whether a previously reported 6mA methyltransferase DAMT-1,^6^ which has been shown to elevate the 6mA level during mitochondrial stress,^18^ is required to establish 6mA marks upon pathogen infection. Compared to the WT animals, *damt-1* KO animals were not able to upregulate 6mA levels under mitochondrial stress (Supplementary information, Fig. S1c, d), consistent with the previous results. However, *damt-1* deficiency did not affect the induction of 6mA under pathogen infection (Supplementary information, Fig. S1e, f). These results indicate that another methyltransferase may function during infection to establish 6mA marks.

### METL-9 is the 6mA methyltransferase that responds to infection

**Fig. 2:**
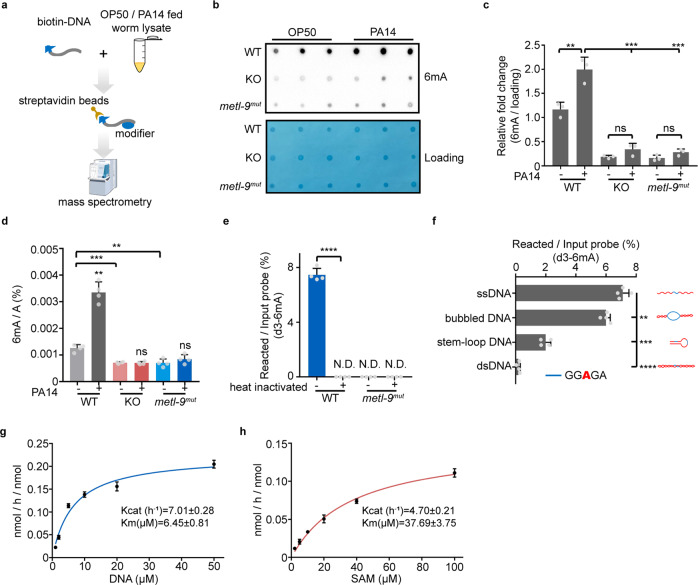
METL-9 is the DNA 6mA methyltransferase responsible for 6mA elevation upon infection. **a** Biotin-DNA pull-down strategy. **b**, **c** 6mA dot blotting (**b**) and quantification (**c**) of WT, *metl-9* KO and *metl-9*^*mut*^ animals fed on OP50 or PA14. *n* = 3. Error bars indicate means + SD. Two-tailed *t*-test, ns, no significance, **P < 0.01, ***P < 0.001. **d** LC-MS/MS analysis of WT, *metl-9* KO and *metl-9*^*mut*^ animals fed on OP50 or PA14. *n* = 4. Error bars indicate means + SD. Two-tailed *t*-test, **P < 0.01, ***P < 0.001. **e** In vitro DNA 6mA methyltransferase activity assay of WT and mutant METL-9 recombinant proteins with deuterated SAM (d3-SAM) and ssDNA substrate. *n* = 4. Error bars indicate means + SD. Two-tailed *t*-test, ****P < 0.0001. **f** DNA substrate preference of METL-9 analyzed by the in vitro methylation assay using d3-SAM. *n* = 4. Error bars indicate means + SD. Two-tailed *t*-test, **P < 0.01, ***P < 0.001, ****P < 0.0001. **g**, **h** Analysis of the kinetic parameters of METL-9 with increasing concentrations of DNA (**g**) or SAM (**h**). METL-9 was incubated with d3-SAM in vitro. *n* = 4. Error bars indicate means + SD.

To identify the methyltransferase that catalyzes gDNA 6mA modifications during infection, we carried out a biotin-labeled DNA oligo pull-down experiment.^26^ The biotin-labeled DNAs, containing the GAGG and AGAA motifs reported in the published 6mA SMRT-seq data from nematodes,^6^ were incubated with total lysate of *C. elegans* cultured in the presence or absence of PA14 (Fig. 2a). Mass spectrometry analysis identified four candidate methyltransferases that associate with the DNA oligos. Among them, METL-9 and H20J04.9 specifically accumulated in the samples extracted from PA14-infected animals (Supplementary information, Fig. S2a). RNA interference (RNAi) knockdown of each candidate revealed that lack of METL-9 decreased the basal 6mA level in *C. elegans* (Supplementary information, Fig. S2b, c). We further employed the CRISPR/Cas9 approach to generate a *metl-9* KO strain. The KO strain has a 101-base pair (bp) insertion in exon 5 of the *metl-9* gene that impairs METL-9 expression (Supplementary information, Fig. S2d–f). KO of *metl-9* decreased the basal gDNA 6mA levels and suppressed the induction of genomic 6mA upon pathogen infection (Fig. 2b–d). In contrast, neither *metl-9* KO nor pathogen infection affected RNA m^6^A modification in *C. elegans* (Supplementary information, Fig. S2g). Overexpression (OE) of METL-9 elevated the basal level of 6mA under normal conditions. The abundance of 6mA did not further increase in METL-9 OE worms after pathogen infection (Supplementary information, Fig. S2h, i). Taken together, these results indicate that METL-9 specifically responds to pathogen infection to establish 6mA marks on the gDNA.

To further verify that METL-9 is the bona fide DNA 6mA methyltransferase, we expressed and purified METL-9 recombinant proteins (Supplementary information, Fig. S3a), and performed in vitro DNA methylation assays using isotope-labeled S-adenosyl methionine (d3-SAM). 6mA methyltransferase activity was detected when DNAs were incubated with WT but not heat-inactivated METL-9 protein (Fig. 2e). Incubating METL-9 protein with DNA substrates with different secondary structures revealed that METL-9 prefers single-stranded DNA (ssDNA), bubbled DNA or stem-loop DNA instead of double-stranded DNA (dsDNA) (Fig. 2f). Moreover, METL-9 displayed no methyltransferase activity towards single-stranded or double-stranded RNA (Supplementary information, Fig. S3b). The substrate preference of METL-9 is consistent with that of the mammalian 6mA demethylase ALKBH1,^27^ and suggests that 6mA methylation may occur in ssDNA or partially uncoiled DNA where deoxyadenine is exposed. We further characterized the enzymatic properties of METL-9 in the presence of an ssDNA substrate or d3-SAM. METL-9 displayed the typical catalytic activity of a methyltransferase, i.e., transferring a methyl group from the SAM donor to the *N*^6^ of adenine (Fig. 2g, h). Notably, the enzyme kinetics of METL-9 are similar to that of the 5mC methyltransferase DNMT1,^28^ supporting the idea that METL-9 is a DNA 6mA methyltransferase in *C. elegans*.

6mA has also been detected in mammalian genomes.^8,21,22,27^ Therefore, we wanted to know whether the mammalian ortholog of *C. elegans* METL-9 displays a conserved 6mA methyltransferase activity. We purified recombinant mouse Mettl9 protein (Supplementary information, Fig. S3c) and performed the in vitro methyltransferase assay. Similar to nematode METL-9, mouse Mettl9 protein showed 6mA methyltransferase activity towards ssDNA and bubbled DNA containing the “GAGG” motif, and a minor activity towards the stem-loop DNA, but no activity towards dsDNA (Supplementary information, Fig. S3d, e). These results suggest that mouse Mettl9 plays a conserved role in serving as a potential DNA 6mA writer in mammals.

We further tested whether mutation of any amino acid of METL-9 would impair its catalytic activity. Twelve residues were predicted by bioinformatics analysis to form a binding pocket for SAM, the methyl donor cofactor required for the methyltransferase activity (Supplementary information, Fig. S4a). We mutated each of the twelve residues and expressed either WT or mutant METL-9 proteins in HEK293T cells. N172K (M8), L173D (M9) or D274G (M10) mutation dramatically impaired the ability of METL-9 to increase 6mA abundance (Supplementary information, Fig. S4b–d). Furthermore, the N172K and D274G double mutant (M8 + M10, *metl-9*^*mut*^) almost completely abolished the 6mA methyltransferase activity of METL-9 (Fig. 2e; Supplementary information, Fig. S4e–h). We employed the CRISPR/Cas9 approach again to generate *metl-9*^*mut*^ animals that harbor the N172K and D274G double mutation. Compared to WT animals, *metl-9*^*mut*^ animals had decreased gDNA 6mA levels under both normal and pathogen-infected conditions, similar to *metl-9* KO animals (Fig. 2b–d). In summary, these results indicate that METL-9 acts as a methyltransferase to establish 6mA marks on the gDNA of animals upon pathogen infection.

### METL-9 is important for the innate immunity of animals against pathogen infection

**Fig. 3:**
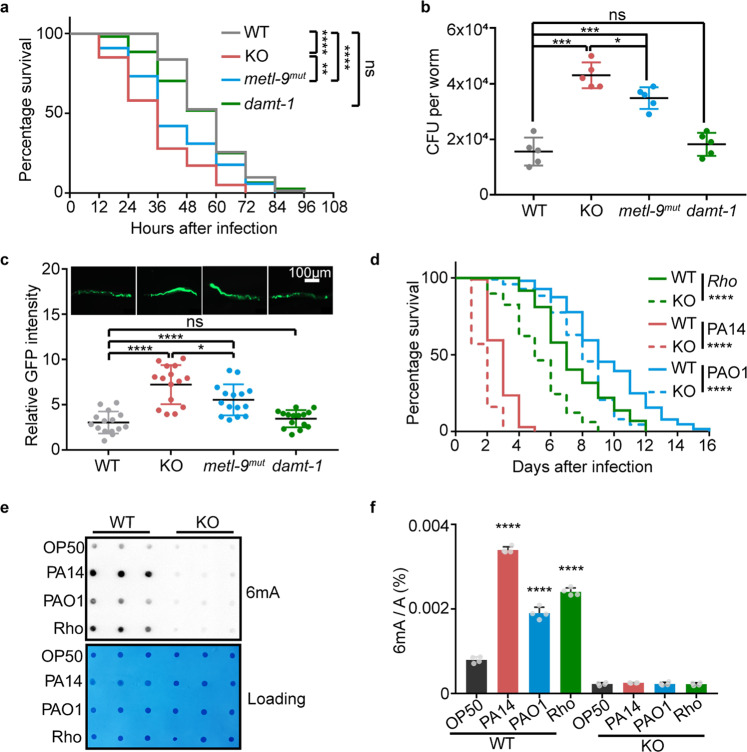
METL-9 is important for the innate immunity of animals against pathogen infection. **a** Slow-killing assay of WT, *metl-9* KO, *metl-9*^*mut*^ and *damt-1* animals fed on PA14. *n* = 100. Log-rank (Mantel–Cox) test, **P < 0.01, ****P < 0.0001. **b** CFU assay for intestinal bacteria in WT, *metl-9* KO, *metl-9*^*mut*^ and *damt-1* animals fed on PA14. *n* = 5. Error bars indicate means + SD. Two-tailed *t*-test, ns, no significance, *P < 0.05, ***P < 0.001. **c** Microscopy and quantification of WT, *metl-9* KO, *metl-9*^*mut*^ and *damt-1* animals fed on PAO1-GFP. *n* = 15. Error bars indicate means + SD. Two-tailed *t*-test, *P < 0.05, ****P < 0.0001. **d** Slow-killing assay of WT and *metl-9* KO animals fed on PA14, PAO1 or *Rhodococcus*. *n* = 100. Log-rank (Mantel–Cox) test, ****P < 0.0001. **e**, **f** 6mA dot blotting (*n* = 3) (**e**) and 6mA LC-MS/MS (*n* = 4) (**f**) of gDNA from WT and *metl-9* KO animals fed with OP50, PA14, PAO1 or *Rhodococcus*. Error bars indicate means + SD. Two-tailed *t*-test, ****P < 0.0001.

After finding that METL-9 catalyzes an increase in 6mA levels upon infection, we were prompted to investigate whether *metl-9* functions to modulate the innate immunity of *C. elegans*. Compared to WT animals, *metl-9* KO and *metl-9*^*mut*^ animals exhibited a shorter survival time and a lower survival rate in the slow-killing assay^29^; the mutant animals also had a higher intestinal colony-forming unit (CFU) count, which indicates more pathogen accumulation due to suppressed host immunity (Fig. 3a–c).^30^ In contrast, deficiency of *damt-1* did not affect the immunity of animals (Fig. 3a–c). Diminished innate immunity and impaired 6mA induction were also observed in *metl-9* KO animals challenged with different pathogens (Fig. 3d–f).

The major source of contamination when studying 6mA in eukaryotes is bacteria,^12,14,16^ which have a high abundance of 6mA modification. *C. elegans* consumes a large number of bacteria as food. Therefore, to exclude the possibility that the detected changes in 6mA levels are due to bacterial contamination, we first employed a bacterial DNA-free approach in which we treated worms with the *Pseudomonas* toxins hemipyocyanine or tubermycin B to induce innate immune responses.^31^ Toxin treatments also induced genomic 6mA levels in *C. elegans* (Supplementary information, Fig. S5a). Moreover, we quantified the ratio of bacterial gDNA contamination in WT, *metl-9* KO and *metl-9* catalytically dead mutant animals fed with OP50 or PA14 bacteria. The degree of bacterial DNA contamination is comparable between all three strains (Supplementary information, Fig. S5b, c). In addition, intestinal CFU counts indicated that the three strains consumed the same amount of bacterial food (Supplementary information, Fig. S5d, e). More importantly, LC-MS/MS revealed that the genomic 6mA level of OP50 is much higher (~23-fold) than that of PA14 (Supplementary information, Fig. S5f). Taken together, these results demonstrated that the elevation of 6mA upon PA14 infection, and the decreased 6mA level in *metl-9* mutant animals, are not due to bacterial contamination.

**Fig. 4:**
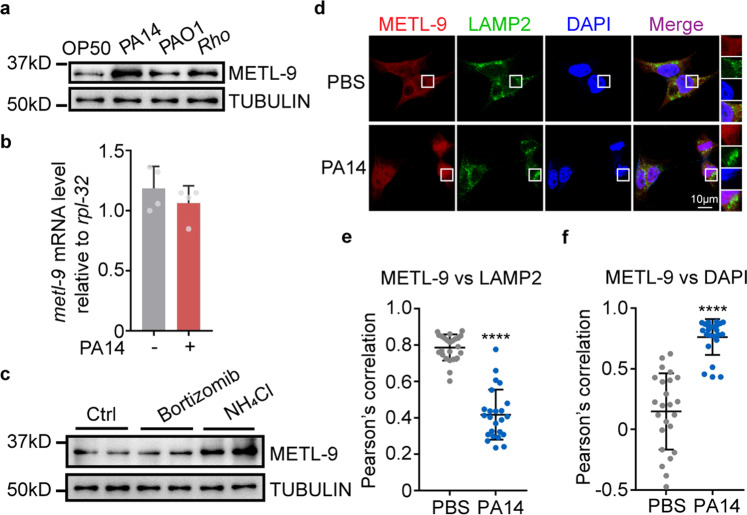
Lysosome-dependent degradation of METL-9 proteins upon infection. **a** METL-9 protein level in WT animals fed with OP50, PA14, PAO1 or *Rhodococcus*. **b** RT-qPCR analysis of the *metl-9* mRNA level in the presence or absence of PA14 infection. Error bars indicate means + SD. *n* = 4. **c** METL-9 protein levels in WT animals treated with proteasome inhibitor bortezomib or lysosome neutralizer NH_4_Cl. *n* = 2. **d**–**f** Immunofluorescence (**d**) and Pearson’s correlation (**e**, **f**) of HA-METL-9, LAMP2 and DAPI in HEK293T cells in the presence or absence of PA14 infection. *n* = 24 (**e**, **f**). Error bars indicate means + SD. Two-tailed *t*-test, ****P < 0.0001.

Interestingly, the METL-9 protein levels were elevated after pathogen infections (Fig. 4a; Supplementary information, Fig. S2f). The induction of METL-9 protein was not due to transcriptional regulation (Fig. 4b). Treating *C. elegans* with the lysosomal acidity neutralizer NH_4_Cl, but not the proteasome inhibitor Bortezomib,^32^ resulted in the accumulation of METL-9 protein (Fig. 4c). We further expressed a low level of *C. elegans* METL-9 in mammalian HEK293T cells and revealed a lysosomal localization of METL-9 under normal conditions (Fig. 4d). More importantly, upon infection, METL-9 displayed a decreased lysosomal localization, but an elevated nuclear localization (Fig. 4d–f). Taken together, these results suggest that pathogen infections might inhibit the lysosome-mediated degradation of METL-9 and promote an innate immune response.

### METL-9 regulates the immune response in 6mA-dependent and -independent manners

To study the mechanism whereby METL-9 controls innate immunity upon pathogen infection, we examined the expression pattern of genes in the WT, *metl-9* KO and *metl-9*^*mut*^ strains in the presence or absence of infection. RNA sequencing (RNA-seq) revealed that the expression patterns of genes in *metl-9* KO and *metl-9*^*mut*^ are similar to each other but different from those of WT animals (Supplementary information, Fig. S6a). 1809 differentially expressed genes (DEGs) were detected upon pathogen infection in WT animals, including 1110 downregulated and 699 upregulated genes. Among them, 947 downregulated and 531 upregulated genes were dependent on the 6mA methyltransferase activity of METL-9 (WT vs *metl-9*^*mut*^) (Supplementary information, Table S1). Gene ontology (GO) analysis of the DEGs revealed that the induction of the defense and immune response pathways required the presence of METL-9 or its 6mA methyltransferase activity (Supplementary information, Fig. S6b–e and Table S2), which suggests that METL-9 and its 6mA methyltransferase activity are required for transcriptional regulation of the innate immune response.

Unraveling the genomic distribution of 6mA sites is critical for the mechanistic study of 6mA-mediated immunomodulation. We further employed SMRT-seq to reveal the genomic 6mA distribution upon infection. SMRT-seq has great potential to identify 6mA modifications at single-nucleotide resolution. However, the usage of SMRT-seq in identifying 6mA sites was previously limited by its high false-positive rate. Through a collaborative study with Zhang et al., we developed an improved method named 6mA-Sniper, which shows promising accuracy in the identification of 6mA modifications at single-nucleotide resolution.^20^ With 6mA-Sniper, Zhang et al. characterized the 6mA profiles in WT and *metl-9* KO animals in the presence or absence of infection, and showed that the basal level of 6mA and the induction of 6mA upon pathogen infection were both impaired in *metl-9* KO animals.^20^ These findings are consistent with the results of dot blotting and LC-MS/MS in this study.

**Fig. 5:**
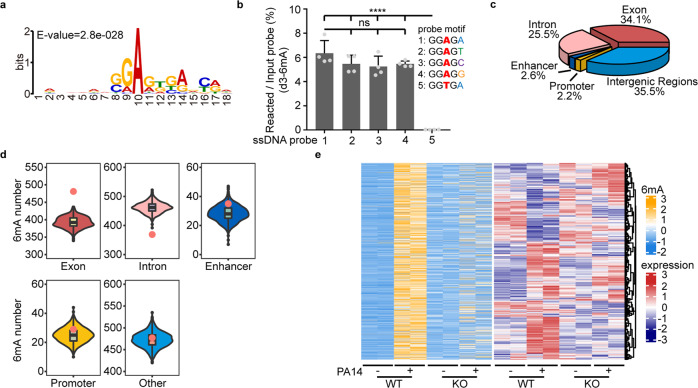
6mA transcriptionally modulates the immune response. **a** Motif analysis showing the frequency of bases surrounding enriched 6mA sites upon PA14 infection in WT animals. The consensus motif is GGAG. **b** DNA motif preference of METL-9 analyzed by the in vitro methylation assay using d3-SAM. *n* = 4. Error bars indicate means + SD. Two-tailed *t*-test, ns, no significance, ****P < 0.0001. **c** Distribution of enriched 6mA sites in different functional categories of gDNA upon PA14 infection in WT animals. **d** Genomic distribution of enriched 6mA sites (red dots) upon PA14 infection compared to an equal number of random non-6mA sites (boxes) in WT animals. **e** Left panel: Heatmaps of the frequency of enriched 6mA sites in gDNA in WT and *metl-9* KO animals upon PA14 infection. Right panel: Heatmaps of the mRNA expression levels of the 6mA-modified genes in WT and *metl-9* KO animals upon PA14 infection. Only genes with FPKM > 2 in at least one of these samples are shown in the heatmap.

Using 6mA-Sniper, we identified 1342 significantly enriched (newly emerged and increased) 6mA sites in WT animals after pathogen infection (Supplementary information, Table S3). These sites were enriched in a “GGAG” motif (Fig. 5a), the same as the one previously reported in *C. elegans*.^6^ We synthesized ssDNA probes with the “GGAG” motif and performed in vitro methylation assays with the use of recombinant METL-9 protein. METL-9 catalyzed DNA methylation when incubated with the DNA probes containing the sequence “GGAGA”. More importantly, mutation of the first adenine to thymine in the “GGAGA” sequence completely abolished the 6mA modification by METL-9, whereas mutation of the second adenine did not affect the methylation activity (Fig. 5b). Consistent with the above results, METL-9 ChIP-seq experiments also identified “GGAG” as one of the preferred binding motifs of METL-9 (Supplementary information, Fig. S6f).

The 1342 significantly enriched 6mA sites upon infection were broadly distributed across different functional types of gDNA (e.g., coding regions, control elements, intergenic regions) in *C. elegans* (Fig. 5c). The 6mA sites were significantly enriched in exonic regions and modestly enriched in enhancers or promoters (Fig. 5d). The enrichment of 6mA sites upon infection was impaired in the *metl-9* KO strain (Fig. 5e). Bioinformatics analysis identified 892 genes that were modified by these 6mA marks. Interestingly, 6mA sites were significantly enriched in upregulated genes (Supplementary information, Fig. S6g). Moreover, Zhang et al. analyzed a group of genome-sequenced *C. elegans* strains and identified 19 single-nucleotide polymorphisms which occurred at genomic 6mA sites and affected the expression of the corresponding genes. Among 10 polymorphisms with significant effects on the expression of the corresponding gene, 9 of them had a down-regulatory effect.^20^ These results together suggest that 6mA might positively regulate gene transcription in *C. elegans*. To directly test the role of 6mA in regulating gene transcription, we mutated SMRT-seq-identified 6mA sites into non-A sites using the CRISPR/Cas9 approach and measured the expression level of the nearby genes before and after infection. Mutation of A (6mA) to G at ChrIV12989886 (–), in the promoter region of *K10D11.6*, greatly impaired the induction of *K10D11.6* transcripts upon infection (Supplementary information, Fig. S6h, i). More cases were tested and similar results were observed in the study of Zhang et al.^20^ In addition, transcriptional regulation of 128 significantly upregulated genes was compromised globally in the *metl-9* KO strain (Fig. 5e; Supplementary information, Table S1). The induction of multiple immune response genes, such as *dct-17*, *gar-2*, *dod22* and *clec* family genes,^33–36^ was significantly suppressed in the *metl-9* KO strain upon infection, compared to the WT strain (Supplementary information, Fig. S6j). Knockdown of *dct-17* or *gar-2* has been shown to impair pathogen resistance in *C. elegans*.^34,35^ In addition, *clec* family genes have also been implicated in modulating *C. elegans* immunity.^36^ Therefore, the reduced expression of these immune response genes in the *metl-9* KO strain results in immune deficiency. Previous studies suggested that 6mA may function in concert with other epigenetic modifications.^6,18,19,22^ ChIP-seq analysis revealed that H3K27me3 signals upstream of 6mA sites increased upon PA14 infection in *metl-9* KO worms, but not in WT animals (Supplementary information, Fig. S6k). Zhang et al. also reported that 6mA is mutually exclusive with H3K27me3 modification, a transcriptionally repressive epigenetic mark,^20^ which suggests a positive role of 6mA in transcriptional regulation. In summary, these results demonstrate that 6mA modifies immune response genes and positively regulates the transcription of these genes upon infection.

**Fig. 6:**
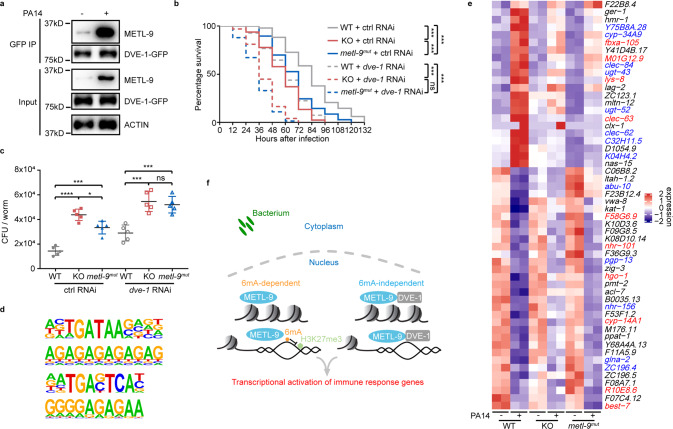
METL-9 interacts with DVE-1 to regulate the immune response. **a** Immunoprecipitation of DVE-1 using anti-GFP antibody in *dve-1p::dve-1::gfp* animals reveals the enhanced interaction of DVE-1 with METL-9 after infection. **b** Slow-killing assay of WT, *metl-9* KO and *metl-9*^*mut*^ nematodes fed with *dve-1* RNAi and challenged with PA14. *n* = 100. Log-rank (Mantel–Cox) test, ns, no significance, ***P < 0.001. **c** CFU assay for intestinal bacteria in WT, *metl-9* KO and *metl-9*^*mut*^ animals fed with *dve-1* RNAi and challenged with PA14. *n* = 5. Error bars indicate means + SD. Two-tailed *t*-test, *P < 0.05, ***P < 0.001, ****P < 0.0001. **d** DVE-1 ChIP-seq analysis reveals a binding preference for “GGAG” or “GAG”, similar to METL-9 and 6mA. **e** Expression heatmap of methyltransferase activity-independent genes. Red text indicates *dve-1*-dependent immune-related genes; blue text indicates *dve-1*-independent immune-related genes. **f** Working model of how METL-9 modulates the innate immune response.

We noticed that the *metl-9*^*mut*^ strain displayed partial resistance to pathogens, compared to the *metl-9* KO animals (Fig. 3a, b). Considering that *metl-9*^*mut*^ almost completely lost its 6mA methyltransferase activity (Fig. 2b–e), the residual pathogen resistance of *metl-9*^*mut*^ animals might be attributed to the non-catalytic activity of METL-9. It has been reported that the RNA m^6^A modifier METTL3 regulates mRNA translation through both catalytic and non-catalytic activities by interacting with the translation initiation factor.^37–39^ We, therefore, hypothesized that METL-9 might modulate innate immunity partially through its physical interaction with other regulators. To search for proteins that interact with METL-9 upon infection, we performed METL-9 immunoprecipitation followed by mass spectrometry analysis. DVE-1, a chromatin organizer protein which functions in the innate immune response,^40,41^ strongly interacted with METL-9 upon pathogen infection (Fig. 6a). More importantly, knockdown of *dve-1* decreased the pathogen resistance of *metl-9*^*mut*^ to the same level as the *metl-9* KO animals (Fig. 6b, c). ChIP-seq analysis revealed that METL-9 and DVE-1 share the same preferred binding sequence containing the “GGAG” or “GAG” motif (Fig. 6d).^41^ RNA-seq analysis revealed that among the 54 genes which depend on METL-9 but not its 6mA methyltransferase activity, 10 genes were also reported to be regulated by DVE-1 (Fig. 6e; Supplementary information, Table S1).^41^ Taken together, these data indicate that a methyltransferase activity-independent function of METL-9 is required to collaborate with DVE-1 and modulate innate immunity.

In summary, our findings revealed that genomic 6mA levels undergo dynamic changes in response to pathogen infection. We further identify METL-9 as the methyltransferase that fine-tunes 6mA levels and modulates innate immunity upon pathogen infection. METL-9 regulates the transcription of the defense and immune response genes through dichotomous mechanisms by catalyzing DNA 6mA modifications and interacting with the chromatin organizer DVE-1 (Fig. 6f).

## Discussion

Recent literature has cast doubts on the existence and physiological function of 6mA in eukaryotes.^15,16^ However, our results reveal that genomic 6mA levels substantially increase in response to pathogen infection. The identification of METL-9 as the infection-regulated 6mA methyltransferase allows us to manipulate the expression level and activity of this enzyme, through genetic and biochemical approaches, and directly affect 6mA abundance. The robust changes of 6mA levels in the *metl-9* KO and *metl-9*^*mut*^ strains, or upon pathogen infection, is not due to bacterial contamination. Furthermore, in vitro kinetic studies reveal that recombinant METL-9 displays DNA methyltransferase properties similar to those reported for the mouse 5mC methyltransferase DNMT1.^28^ The catalytic efficiency of METL-9 is lower than that of the *E. coli* 6mA methyltransferase Dam (METL-9: *K*_cat_ (h^−1^) = 7.01 ± 0.28 vs Dam: *K*_cat_ (h^−1^) = 540 ± 72).^42^ This finding correlates with the observation that the percentage of 6mA in the *C. elegans* genome is much less than that in the *E. coli* genome (0.001% – 0.002% vs 0.6% – 0.8%), and it takes a few hours for the worms to elevate their genomic 6mA levels by 3 – 4-fold after infection. Moreover, the “GGAG” motif identified by the 6mA-Sniper analysis and METL-9 ChIP-seq analysis, and verified by the in vitro biochemical assay argues that genomic 6mA in the *C. elegans* genome is not accidentally incorporated as a result of the RNA m^6^A metabolism.

Mouse Mettl9 has been reported to function as a protein histidine methyltransferase.^43–46^ The current study indicates that mouse Mettl9 also shows DNA 6mA methyltransferase activity in vitro. The weaker activity of mouse Mettl9 observed in this study may reflect the intrinsic enzymatic properties, or it may result from imperfectly optimized purification or substrate specificity. These results suggest that Mettl9 may work with different substrates to modulate various biological processes. Based on our results, we feel that the existence and potential function of DNA 6mA in eukaryotes should be re-evaluated. Whether the level of 6mA changes when animals or cells are challenged with different stresses, and whether 6mA plays a role in modulating stress responses warrant further investigation.

Using an improved single-nucleotide resolution sequencing method, 6mA-Sniper, we and our colleagues Zhang et al. characterized the 6mA profiles in WT or *metl-9* KO strains in the presence or absence of the infection, and uncovered the important function of 6mA in transcriptional regulation. CRISPR/Cas9-mediated mutation of SMRT-seq-identified 6mA sites into non-A bases revealed a reduced level of gene expression, further suggesting that 6mA may modulate gene transcription. Induction of 6mA upon pathogen infection affects the expression of a group of immune response genes, indicating that 6mA is a dynamically regulated DNA modification, which is functionally important to help worms cope with pathogen infection.

In the future, it will be important to explore the detailed mechanisms underlying the modulatory effect of 6mA on transcription. Further analysis is required to determine whether 6mA coordinates with histone methylation or other epigenetic modifications, such as H3K27me3.^6,18,19,22^ For instance, it will be interesting to test whether 6mA plays a negative role in PRC2 recruitment. Moreover, in the joint study with Zhang et al., we found that 6mA sites are preferentially located within nucleosomes.^20^ Considering that the 6mA methyltransferase METL-9 interacts with the chromatin organizer DVE-1, further explorations are warranted to investigate whether 6mA functions in chromatin remodeling. It will also be important to understand whether 6mA only affects the expression of nearby genes, or whether it participates in long-distance regulation through reorganization of the 3D genome structure.

## Materials and methods

### *C. elegans* strains and culture

Worm strains used in this study are listed in Supplementary information, Table S5. Animals were maintained on nematode growth medium (NGM) at 20 °C and fed on *E. coli* OP50, or transferred to other media or buffers where indicated.

### Generation of worm strains

*metl-9*^*mut*^(*syb2872*) was generated by SunyBiotech using the CRISPR/Cas9 approach. Two sgRNAs and a donor sequence with the N172K (AAC to AAG) and D274G (GAC to GGC) mutations were used to introduce point mutations in the *T03G11.6* (*metl-9*) locus. The FLAG-METL-9 OE strain was generated by injection of *rpl-28p*::flag-*metl-9* and *odr-7p*::dsRED plasmids into the *metl-9* KO strain. The *metl-9* KO strain was generated by our lab using the CRISPR/Cas9 approach. sgRNAs and donor sequences for the above-mentioned strains are listed in Supplementary information, Table S4.

### Bacterial strains

OP50, RNAi bacteria, PA14, *ΔgacA* PA14 and *Rhodococcus* are in-house resources. PAO1-GFP was a gift from Dr. Huanqin Dai.

### Genotyping

Ten animals were picked into 10 μL of genotyping buffer (10 mM Tris-HCl, pH 8.0, 50 mM KCl, 2.5 mM MgCl_2_, 0.45% IGEPAL CA-630, 0.45% Tween-20, 0.2 mg/mL proteinase K), incubated at 65 °C for 1 h, and then denatured at 95 °C for 5 min. 2 μL supernatant was used for PCR analysis. Primer sequences for genotyping are shown in Supplementary information, Table S4.

### Antimycin treatment

Synchronized animals were seeded to OP50-NGM plates with or without 1.92 μM antimycin A, and grown at 20 °C until L3–L4 stage. Animals were collected and washed with M9 buffer (42.3 mM Na_2_HPO_4_, 22.1 mM KH_2_PO_4_, 85.5 mM NaCl) before gDNA extraction.

### Hemipyocyanine and tubermycin B treatment

L3–L4 animals were washed off from gDNA plates with M9 buffer and transferred to OP50-seeded NGM plates with hemipyocyanine (hydroxyphenazine, 76 μM), tubermycin B (phenazine-1-carboxylic acid, 67 μM) or DMSO. Plates were kept at 20 °C for 3 h. Samples were collected and washed with M9 before gDNA extraction.^31^

### Bortezomib and NH_4_Cl treatment

L3–L4 animals were washed off from plates with M9 buffer. Animals were resuspended with M9 buffer containing OP50 and drug (5 μg/mL bortezomib or 200 mM NH_4_Cl) and rotated at 20 °C for 8 h (bortezomib) or 4 h (NH_4_Cl) before they were collected.^32^

### Pathogen infection (except CFU assay, slow-killing assay and PAO1-GFP accumulation)

Pathogens were grown in LB and seeded onto slow-killing plates (*Pseudomonas* was grown in carbenicillin-LB at 37 °C overnight; *Rhodococcus* was grown in LB at room temperature for 2 days). Plates were air-dried. The pathogens were grown in the plates for 48 h (*Pseudomonas* at 37 °C for 24 h and then another 24 h at room temperature; *Rhodococcus* at room temperature for 48 h).^30^ The plates were then transferred to 20 °C and incubated for 1 h before use. L4 animals were transferred to pathogen plates and cultured at 20 °C for 2 h. Animals were then collected in M9 buffer.

### Slow-killing assay

Pathogen plates were prepared as described above. Animals were fed with control or indicated RNAi bacteria until they reached the L4 stage. One hundred animals were randomly picked onto a slow-killing plate containing the pathogen. Live animals were counted every day or every 12 h. Live animals were transferred carefully to new pathogen plates every 2 days. Animals that died due to ruptured vulvae, internal hatching or crawling off the agar were removed.

### CFU assay

Animals were challenged with PA14 under the same conditions as in the slow-killing assay. After 48 h, five replicates of ten animals were randomly picked into M9 buffer containing 25 mM levamisole. Animals were washed three times with M9 buffer containing 1 mg/mL carbenicillin, 1 mg/mL gentamicin and 25 mM levamisole, followed by rotation in antibiotic buffer for 1 h to kill external bacteria on the surface of animals. Three washes with M9 buffer containing 25 mM levamisole were then performed to remove the antibiotics. Animals were resuspended in 100 μL saline and ground with a motorized pestle. Worm lysates were serially diluted 10 times. For each dilution, triplicates of 10 μL suspension were dropped onto LB plates containing 50 µg/mL of carbenicillin. After overnight cultivation at 37 °C, the colonies were counted.^29^

### Intestinal PAO1-GFP accumulation

PAO1-GFP plates were prepared as described above. PAO1-GFP is a *Pseudomonas aeruginosa* strain stably expressing GFP. Animals were challenged with PAO1-GFP under the same conditions as in the slow-killing assay. After 48 h, animals were randomly picked and imaged using a Carl Zeiss Axio Imager M2.^30^ Quantification of fluorescence was performed by ImageJ.

### RNAi

*C. elegans* RNAi bacteria were obtained from the Ahringer library (Source Bioscience, Nottingham, UK). *metl-9* and *H20J04.9* RNAi bacteria were generated by us with the primer sequences in Supplementary information, Table S4. All RNAi plasmids were confirmed by Sanger sequencing. RNAi knockdown efficiencies were verified by RT-qPCR using the indicated primer sets in Supplementary information, Table S4. RNAi clones were grown in LB containing 50 μg/mL carbenicillin at 37 °C overnight and seeded onto NGM plates with 2.4 mM IPTG. Dried plates were kept at room temperature overnight to allow IPTG induction of dsRNA expression before animals were seeded.

### gDNA extraction

Animals were washed off from plates with M9 buffer. After 4 additional washes with M9 buffer (total time: 0.5 h), worm pellets were resuspended with at least 5 volumes of worm gDNA lysis buffer (200 mM NaCl, 100 mM Tris-HCl, pH 8.5, 50 mM EDTA, pH 8.0, 0.5% SDS) with 0.1 mg/mL freshly added proteinase K. The mixture was incubated at 55 °C with several gentle mixes until clear. 0.5 mg/mL RNase A was added and incubated at 37 °C for 1 h. gDNA was isolated by phenol:chloroform:isopentanol (25:24:1) extraction, followed by acetate and isopropanol precipitation. The pellet was washed with 70% ethanol, air-dried and resuspended in TE buffer (10 mM Tris-HCl, pH 8.0, 1 mM EDTA, pH 8.0). 1/20 volume of RNase A/T1 and 1/50 volume of RNase H were added into the solution and incubated at 37 °C for 1 h, followed by gDNA re-purification starting from the phenol:chloroform:isopentanol extraction step.^18^ For OP50 or PA14 gDNA, overnight cultured bacteria were collected and washed 2 times with PBS, and then gDNA was extracted following the worm gDNA extraction protocol.

### 6mA dot blotting

Worm gDNA was denatured at 95 °C for 10 min and chilled on ice for 2 min. Hybond-N+ membrane was pre-baked at 80 °C for 10 min. 300 ng DNA was loaded per dot onto the membrane. The membrane was baked at 80 °C for another 1 h followed by auto-crosslinking twice in a UVP CL-1000 Ultraviolet Crosslinker, with 0.15 J/cm^2^ energy density. After being blocked with 5% non-fat milk in TBST, the membrane was probed with anti-6mA antibody and secondary antibody, and visualized by a Tanon 5200 chemical luminescence imaging system. After imaging, the membrane was stained by methylene blue solution to visualize DNA loading.^16^ Quantification of 6mA dot blotting was performed by Image J. The 6mA signal was normalized to the loading signal.

### Immunoblotting

After the indicated treatment and worm collection, the freshly pelleted animals were lysed in RIPA buffer with a glass homogenizer. Worm lysates were centrifuged at 4 °C for 15 min. The protein in the supernatant was quantified by the BCA kit. 30 μg total lysate was loaded into an SDS-PAGE gel, followed by electrophoresis and transfer. After being blocked with 5% non-fat milk in TBST, the membrane was probed with the designated primary and secondary antibodies, and visualized by a Tanon 5200 chemical luminescence imaging system. Proteinase inhibitor cocktails were added to all the buffers before use.

### Biotin-labeled DNA pull-down

The animals were challenged with OP50 or PA14 as mentioned above and washed off from plates by M9 buffer to remove bacteria. Freshly pelleted animals were lysed in RIPA buffer with a glass homogenizer. Protein levels in worm lysates were quantified by BCA kit and adjusted to 2.5 mg/mL. 2 μg biotin-DNA was added into 5 mg total lysate and incubated for 2 h at 4 °C with gentle rotation. Incubation of worm lysate with biotin-free DNA was used as control. Dynabead MyOne Streptavidin C1 beads were pre-blocked for 1 h at 4 °C with IP blocking buffer (0.2 mg/mL sonicated salmon sperm DNA, 0.5 mg/mL BSA in TE). After incubation, the reaction was equally transferred to 4 wells of a 6-well cell culture plate. The plate was placed on ice and autocrosslinked twice in a UVP CL-1000 Ultraviolet Crosslinker, with 0.1 J/cm^2^ energy density. 10 μL pre-blocked streptavidin beads were added into the crosslinked reaction and incubated at 4 °C for 30 min with gentle rotation. After pull-down, the beads were washed 6 times with RIPA buffer and 1 time with TE, and then boiled at 95 °C in 20 μL biotin lysis buffer (TE with 1% SDS, 1 mM biotin) for 20 min. Two biotin-free DNA pull-down samples were combined as the negative control. Silver staining and mass spectrometry analysis were then performed to identify the associated proteins. Proteinase inhibitor cocktails were added into RIPA buffer before use.^26^

### Mass spectrometry analysis

Protein bands on the SDS-PAGE gel were de-stained and in-gel digested with sequencing grade trypsin (10 ng/μL trypsin, 50 mM ammonium bicarbonate, pH 8.0) overnight at 37 °C. Peptides were extracted with 5% formic acid/50% acetonitrile and 0.1% formic acid/75% acetonitrile sequentially and then concentrated to a volume of ~20 μL. The extracted peptides were separated by an analytical capillary column (50 μm × 15 cm) packed with 5 μm spherical C18 reversed phase material (YMC, Kyoyo, Japan). A Waters nanoAcquity UPLC system (Waters, Milford, USA) was used to generate the following HPLC gradient: 0%–30% B in 60 min, 30%–70% B in 15 min (A = 0.1% formic acid in water, B = 0.1% formic acid in acetonitrile). The eluted peptides were sprayed into a Q Exactive mass spectrometer (Thermo Fisher Scientific, San Jose, CA, USA) equipped with a nano-ESI ion source. The mass spectrometer was operated in data-dependent mode with one MS scan followed by ten HCD (High-energy Collisional Dissociation) MS/MS scans for each cycle. Database searches were performed on an in-house Mascot server (Matrix Science Ltd., London, UK) against the NCBI *Caenorhabditis elegans* protein database. The search parameters were: 10 ppm mass tolerance for precursor ions; 0.02 Da mass tolerance for product ions; two missed cleavage sites were allowed for trypsin digestion. Methionine oxidation was set as variable modification. The search results were filtered with both peptide significance threshold and expectation value to be below 0.05.

### Purification of GST-METL-9 or GST-Mettl9 protein

The coding sequence of *C. elegans* METL-9 or mouse Mettl9 was inserted into pGEX-Tev vector and sequenced. The sequence-verified plasmid was transformed into BL21 (DE3). The bacteria were cultured in ampicillin-LB at 37 °C until OD600 reached 0.5. The culture was cooled to 16 °C before 0.5 mM IPTG was added. The bacterial culture was shaken at 16 °C for 16 h. The bacteria were then pelleted and washed once by pre-chilled PBS. The pellet was resuspended in buffer T (20 mM Tris-HCl, pH 8.0, 50 mM NaCl) and sonicated. After centrifugation, the supernatant was incubated with Glutathione Sepharose 4B for 3 h (GST-METL-9) or 2 h (GST-Mettl9) at 4 °C with gentle rotation. The Sepharose beads were then washed and pre-eluted sequentially with 10 volumes of high salt buffer T (20 mM Tris-HCl, pH 8.0, 1 M NaCl), 5 volumes of buffer T, 5 volumes of pre-elution buffer I (20 mM Tris-HCl, pH 8.0, 100 mM NaCl), 2 volumes of pre-elution buffer II (20 mM Tris-HCl, pH 8.0, 100 mM NaCl, 2.5 mM L-glutathione), and 2 volumes of pre-elution buffer III (20 mM Tris-HCl, pH 8.0, 100 mM NaCl, 5 mM L-glutathione). Formal elution was then carried out 4 times with 2 volumes of elution buffer (20 mM Tris-HCl, pH 8.0, 100 mM NaCl, 20 mM L-glutathione). The eluate was analyzed by Coomassie blue staining and dialyzed to remove L-glutathione. Proteinase inhibitor cocktails were added to all buffers before use.

### Prediction of ligand-binding sites

The ligand-binding sites were predicted by PointSite,^47^ a deep learning-based algorithm. Different from the previous pocket-centric method, PointSite is a protein-centric method and predicts the per-atom ligand-binding probability. The input protein 3D structure is first transferred to point clouds and then is segmented through Submanifold Sparse Convolution (SSC)-based UNet to obtain the per-atom probabilities.^48^ To better capture the minority ligand-binding points, SSCN maintains the sparsity of the input points (protein atoms) within each convolutional layer, and labels them to be either active or inactive. Meanwhile, by introducing incrementally increased receptive fields and skip connections, the model explicitly considers the connectivity and physicochemical features of the atoms, thus capturing both the local and global geometric information. PointSite is trained on a subset of scPDB (created in 2017)^49^ and is tested on several independent test sets and a blind test set from the CAMEO dataset.^50^ In practice, given an input protein 3D structure (either from an X-ray structure or a predicted 3D structure), PointSite predicts the probabilities of the protein atoms being ligand-binding atoms. Atoms with higher probabilities thus tend to interact with a small molecule. The model can be accessed through github repo: https://github.com/PointSite/PointSite.git.

### In vitro 6mA methyltransferase activity assay

Recombinant GST-METL-9, GST-METL-9^*mut*^ or GST-Mettl9 proteins were incubated with biotin-labeled DNA oligos in the following 20 μL reaction: 15 mM HEPES, pH 7.5, 4% glycerol, 80 mM KCl, 2.5 mM MgCl_2_, 0.2 mM dithiothreitol, 0.16 nmol recombinant protein, 2 μM biotin-oligo, 0.1 mM SAM or d3-SAM as indicated. Heat-inactivated recombinant protein was used as control. The reactions were incubated at 20 °C (*C. elegans* METL-9) or 25 °C (mouse Mettl9) for 2 h. Then ddH_2_O was added to a final volume of 200 μL. 10 μL pre-blocked Dynabead MyOne Streptavidin C1 beads were then added. The mixture was incubated at 25 °C for 0.5 h with gentle shaking. After 5 washes with TE, oligos were digested on beads by DNase I at 37 °C for 2 h with gentle shaking. The supernatant was then treated with nuclease P1 and phosphatase rSAP as described in the 6mA LC-MS/MS method (below). 6mA LC-MS/MS was then performed for quantification. Before reaction, ssDNA or ssRNA oligo was denatured at 90 °C for 5 min and chilled on ice; other oligos were denatured at 90 °C for 5 min and then subjected to programmed cooling to 16 °C at –0.5 °C/min.^51^ For enzyme kinetics measurement with varying amounts of DNA substrate, ssDNA was added at the indicated concentration while d3-SAM was held at 0.1 mM. For enzyme kinetics measurement with varying amount of SAM as substrate, d3-SAM was added at the indicated concentration while ssDNA was held at 5 μM. Reacted probe/Input probe (%) = percentage of 6mA/A × number of A in probe.

### FLAG-tagged WT and mutant METL-9 expression in 293T cells

HEK293T cells were cultured in DMEM high glucose medium supplemented with 10% FBS, penicillin/streptomycin, and Zell Shield. 18 h before transfection, HEK293T cells were plated to 50% confluence in 6-well plates. 1 µg of WT or mutant FLAG-METL-9 plasmid, prepared from *dam*^−/−^ *E. coli*, was transfected by polyethyleneimine. An equal amount of empty vector was transfected into HEK293T cells as control. 60 h after transfection, cells were collected and lysed by RIPA buffer. 15 μg total lysate was used for anti-FLAG immunoblotting. Proteinase inhibitor cocktails were added into RIPA buffer before use.

### Quantification of bacterial contamination by qPCR and CFU

Synchronized nematodes were fed on OP50 at 20 °C until L4, then transferred to slow-killing plates with OP50 or PA14 and fed for another 2 h as described. Animals were washed and collected. gDNA was extracted as described. qPCR was performed on gDNA of N2, *metl-9* KO or *metl-9*^*mut*^ in the presence or absence of PA14 infection using OP50-specific, PA14-specific or *rpl-32*-specific primers. The Cq value was converted into the copy number of bacterial gDNA based on the standard curve for each bacterial sequence. The standard curve for the OP50-specific, PA14-specific or *rpl-32*-specific sequence was generated by qPCR using a plasmid containing each sequence. Four replicates of 10 animals were used for intestinal CFU determination as described above.

### GFP immunoprecipitation

Fresh worm pellets were lysed in RIPA buffer with a glass homogenizer. Worm lysates were centrifuged at 4 °C for 15 min. The protein in the supernatant was quantified by BCA kit and adjusted to 2 mg/mL. 25 μL pre-blocked GFP-Trap agarose was added into 4 mg total lysate. After 6 h rotation at 4 °C, the agarose was washed 5 times with RIPA buffer, and boiled at 95 °C in sample buffer. Immunoblotting with anti-GFP and anti-METL-9 antibodies was then performed. Proteinase inhibitor cocktails were added into RIPA buffer before use.

### Immunofluorescence analysis of HA-tagged METL-9

300 ng of METL-9-HA plasmid was transfected into HEK293T cells in 10-cm dishes with poly-lysine-coated glass slides. 36 h after transfection, cells were treated with 1 mg/mL PA14 total lysate or PBS. 2 h later, cells were washed twice with cold PBS, then fixed by 4% PFA for 20 min at 25 °C. Fixed cells were washed 3 times with PBS, then treated with 0.1% Triton X-100 for 10 min. After 3 washes with PBS, cells were blocked with 5% BSA for 1.5 h at 25 °C. Primary antibodies were added and incubated for 4 h at 25 °C (1:300 for HA antibody, 1:300 for LAMP2 antibody in 5% BSA). Then 4 washes with PBS were performed before 1 h incubation with Alexa Fluor 594 goat anti-rabbit IgG and Alexa Fluor 488 goat anti-mouse IgG (1:1000 in 5% BSA). After 6 more washes with PBS, mounting medium with DAPI was added. The slides were imaged using a Carl Zeiss LSM710 Confocal Microscope with a 63× 1.4 NA oil objective. The images shown are single slices. Colocalization of METL-9-HA with LAMP2 or DAPI was quantitated by ImageJ.

### RNA extraction and RT-qPCR

Worm pellets were washed with M9 buffer and resuspended with TRIzol, then frozen in liquid nitrogen and homogenized. Total RNA was isolated by chloroform extraction, followed by isopropanol precipitation. cDNA was then synthesized by a reverse transcription kit. RT-qPCR was carried out using SYBR GREEN PCR Master Mix. RT-qPCR was performed by using primer sets in Supplementary information, Table S4.

### ChIP-seq

Animals were treated and collected as described above. After sample collection, the worm pellet was suspended in ChIP crosslinking buffer (PBS with 1% formaldehyde) and rotated at room temperature for 20 min. 2/5 volume of 2.5 M glycine was added to stop the reaction. The pellet was washed 3 times with PBS, resuspended in ChIP SDS lysis buffer (1% SDS, 10 mM EDTA, 50 mM Tris, pH 8.1) with protease inhibitor cocktail, homogenized and incubated on ice for 20 min, then sonicated by a SONICS Vibra Cell sonicator. After centrifugation, the supernatant was diluted with ChIP dilution buffer (0.01% SDS, 1.1% Triton X-100, 1.2 mM EDTA, 16.7 mM Tris-HCl, pH 8.1, 167 mM NaCl). 100 μL of pre-blocked Protein A agarose was added and rotated at 4 °C for 1 h. The antibody (anti-H3K27me3: 6 μg per 30 μg chromatin; anti-FLAG: 2 μg per 30 μg chromatin) was then added to the supernatant and rotated at 4 °C for 16 h. 25 μL of pre-blocked Dynabeads Protein G was added and rotated at 4 °C for 2 h. The beads were washed sequentially with the following buffers: low-salt wash buffer (0.1% SDS, 1% Triton X-100, 2 mM EDTA, 20 mM Tris-HCl, pH 8.1, 150 mM NaCl), high-salt wash buffer (0.1% SDS, 1% Triton X-100, 2 mM EDTA, 20 mM Tris-HCl, pH 8.1, 500 mM NaCl), and LiCl wash buffer (250 mM LiCl, 1% IGEPAL-CA630, 1% DOC). After another wash with TE buffer (Tris-EDTA, pH 8.0), the beads were eluted with 250 μL elution buffer (1% SDS, 100 mM NaHCO_3_) for 2 times with a total time of 40 min. 20 μL of 5 M NaCl was added into the eluent, and incubated at 65 °C for 5 h. 20 μL of 1 M Tris-HCl, pH 6.5, 10 μL of 0.5 M EDTA and 20 μg proteinase K were added and incubated at 45 °C for 2 h. DNA was purified by Zymo ChIP DNA Clean and Concentrator. Purified DNA was used for DNA library construction.

### ChIP-seq data analysis

For the ChIP-seq data of H3K27me3 and Flag-METL-9, after quality control by FastQC (v0.11.9), the raw reads were first mapped to the *C. elegans* genome (ce11) by BWA (v0.7.13-r1126) with default parameters.^52^ Properly paired, uniquely-mapped reads with mapping quality ≥ 20 were retained for the following analysis. PCR duplicates were removed by Picard (v2.17.6). Epic2 was used to obtain peak regions of H3K27me3 while MACS2 was used to obtain peak regions of METL-9.^53,54^ Finally, deeptools (v2.4.2) was used to calculate ChIP-seq signals by normalizing the read coverage to 10 million reads then subtracting input coverage from the ChIP samples to get final ChIP-seq signals.^55^

### LC-MS/MS quantification of DNA 6mA

200 ng of DNA substrate was digested in a 25 μL reaction with nuclease-free H_2_O and 1 μL DNase I at 37 °C for 8 h, denatured at 95 °C for 5 min, chilled on ice and then incubated with 3 μL of 100 mM NH_4_OAc, pH 5.3, 1 μL of nuclease P1 and 1 μL ddH_2_O overnight at 42 °C. After the addition of 4 μL of 1 M NH_4_HCO_3_, 1 μL of rSAP and 5 μL of ddH_2_O, the samples were incubated at 37 °C for 8 h. The final solution was centrifuged at 15,000 rpm for 30 min. LC-MS/MS measurements of 2′-deoxyadenosine and *N*^6^-methyl-2′-deoxyadenosine were conducted on a Waters I-Class UPLC coupled with a Xevo TQ-S microMass Spectrometer equipped with an electrospray ionization source (Waters Technologies, Milford, USA). 1 μL digested DNA sample was injected into a Waters ACQUITY UPLC HSS T3, 2.1 × 100 mm, 1.8 μm column (Waters Technologies, Milford, USA) for separation. The column temperature was controlled at 30 °C. Mobile phase A was H_2_O containing 0.1% formic acid, and mobile phase B was acetonitrile also containing 0.1% formic acid. The flow rate was set at 0.4 mL/min. During the whole analysis, the gradient was: 0–1 min, 0% B; 1.5 min, 3% B; 2 min, 5% B; 3–5 min, 8% B; 5–6.5 min, 100% B; 6.5–8 min, 0% B. The autosampler temperature was maintained at 4 °C to avoid sample degradation. The MRM detection of target compounds was performed in positive electrospray ionization (ESI+) mode. The ion source temperature and capillary voltage were kept constant and set to 150 °C and 2.0 kV, respectively. The cone gas flow rate was 20 L/h and the desolvation temperature was 400 °C. For the transition of m/z 252.0 → 136.0 (2′-deoxyadenosine), the cone voltage was 16 V and the collision energy was 10 V; for the transition of m/z 266.0 → 150.0 (*N*^6^methyl-2′-deoxyadenosine), the values were 8 V and 10 V, respectively.^18,56,57^

### LC-MS/MS quantification of RNA m^6^A

200 ng RNA was digested by 1 μL nuclease P1 (Wako) in a 25 μL reaction containing 25 mM NaCl and 2.5 mM ZnCl_2_ at 42 °C for 4 h, followed by the addition of NH_4_HCO_3_ (1 M, 3 μL) and 1 μL rSAP (NEB) and incubation at 37 °C for 2 h. The sample was then centrifuged and injected into an LC-MS/MS system. LC-MS/MS measurements of adenosine and *N*^6^-methyl-adenosine were conducted on a Waters I-Class UPLC coupled with a Xevo TQ-S micro Mass Spectrometer equipped with an electrospray ionization source (Waters Technologies, Milford, USA). 1 μL digested RNA sample was injected into a Waters ACQUITY UPLC HSS T3, 2.1 × 100 mm, 1.8 μm column (Waters Technologies, Milford, USA) for separation. The column temperature was controlled at 30 °C. Mobile phase A was H_2_O containing 0.1% formic acid, and mobile phase B was acetonitrile also containing 0.1% formic acid. The flow rate was set at 0.4 mL/min. During the whole analysis, the gradient was: 0–1 min, 0% B; 1.5 min, 3% B; 2 min, 5% B; 3–5 min, 8% B; 5–6.5 min, 100% B; 6.5–8 min, 0% B. The autosampler temperature was maintained at 4 °C to avoid sample degradation. The MRM detection of target compounds was performed in ESI+ mode. The ion source temperature and capillary voltage were kept constant and set to 150 °C and 2.0 kV, respectively. The cone gas flow rate was 20 L/h and the desolvation temperature was 400 °C. For the transitions of m/z 268.1 → 136.0 (adenosine) and 282.1 → 150.0 (*N*^6^-methyl-adenosine), the cone voltage and collision energy were both set as 20 V.^51,56,57^

### RNA-seq analysis

RNA-seq data were analyzed as previously described. In brief, raw sequencing data were aligned to the *C. elegans* genome reference (ce11) by HISAT2 (v2.2.1) with default settings.^58^ After the low-quality reads were filtered out by samtools (v1.12, -f 34 -F 1564 or -f 18 -F 1580) and sambamba (v0.8.2),^59,60^ fragments mapped on the exons were processed using featureCounts (v2.0.2) to obtain the gene expression matrix.^61^ Gene expression level was generated using edgeR (v3.34.1).^62^ DEGs were analyzed using DESeq2^63^: genes with an expression fold changes > 1.5 and adjusted *P* < 0.05 were considered as DEGs. The GO pathway enrichment analysis was performed using clusterProfiler (v4.2.2).^64^ To distinguish between *metl-9*-dependent and -independent DEGs, genes were compared between WT and *metl-9* KO groups. *metl-9*-dependent DEGs were identified as genes that met one of these criteria: (1) differentially expressed in the WT group but not the *metl-9* KO group; (2) differentially expressed in the WT group and the absolute value of (log_2_FC_WT DEGs_ – log_2_FC_KO DEGs_) > log_2_(1.5).

### Identification and characterization of 6mA sites

The 6mA sites enriched after PA14 infection were identified by 6mA-Sniper as described in Zhang et al.^20^ Briefly, the enriched 6mA sites after PA14 infection were defined as sites that met one of these criteria: (1) newly emerged after infection (supported by more than one significant CCS read in each biological replicate of WT samples treated with PA14) but not detected in any WT samples without PA14 treatment; (2) 6mA level increased after infection (supported by at least five significant CCS reads in WT samples treated with PA14, but only one significant CCS read was detected in WT samples without PA14 treatment). Due to the relatively low sequencing depth in each biological replicate, the SMRT-seq reads of replicates were combined to define sites with an increased 6mA level. The frequency of low-coverage sites (i.e., the sites covered by less than 5 reads in the sample) was assigned as missing data in the calculation. The motif analysis was performed by MEME (v5.4.1, minimum width 3, maximum width 20).^65^ For the distribution analysis of 6mA sites, the exonic, intronic and intergenic regions were defined by the annotation files (WBcel235.87). The enhancer and promoter regions were defined according to the regulatory annotation of Janes et al.^66^ To calculate the distribution of non-6mA sites in the different genomic regions, the same number of sites (equal to the number of enriched 6mA sites upon PA14 infection in WT) were randomly selected 1000 times from the non-6mA sites. In the frequency analysis, the frequency of low-coverage sites (the sites covered by less than 5 reads in the sample) was assigned as missing data in the calculation.

### Quantifications and statistical analysis

Statistical analysis was performed in GraphPad Prism (GraphPad Software Inc.). Experiments yielding quantitative data for statistical analysis were performed independently at least three times with similar results. Micrographs are representative images of at least two independent experiments, with similar results.

## Supplementary information

Supplementary information, Fig. S1

Supplementary information, Fig. S2

Supplementary information, Fig. S3

Supplementary information, Fig. S4

Supplementary information, Fig. S5

Supplementary information, Fig. S6

Supplementary information, Table S1

Supplementary information, Table S2

Supplementary information, Table S3

Supplementary information, Table S4

Supplementary information, Table S5

## Data Availability

Raw sequencing data are available under GEO, RNA-seq: GSE192901, SMRT-seq and ChIP-seq: PRJNA857919.
