# Supplementary material for: A novel N6-Deoxyadenine methyltransferase METL-9 modulates C. elegans immunity via dichotomous mechanisms
Source: Cell Res. 2023 Jun 5;33(8):628–39. doi: 10.1038/s41422-023-00826-y (PMC10397248; doi:10.1038/s41422-023-00826-y)
Supplement: Supplementary file 1 — Supplementary information, Fig. S1 [file 41422_2023_826_MOESM1_ESM.pdf]

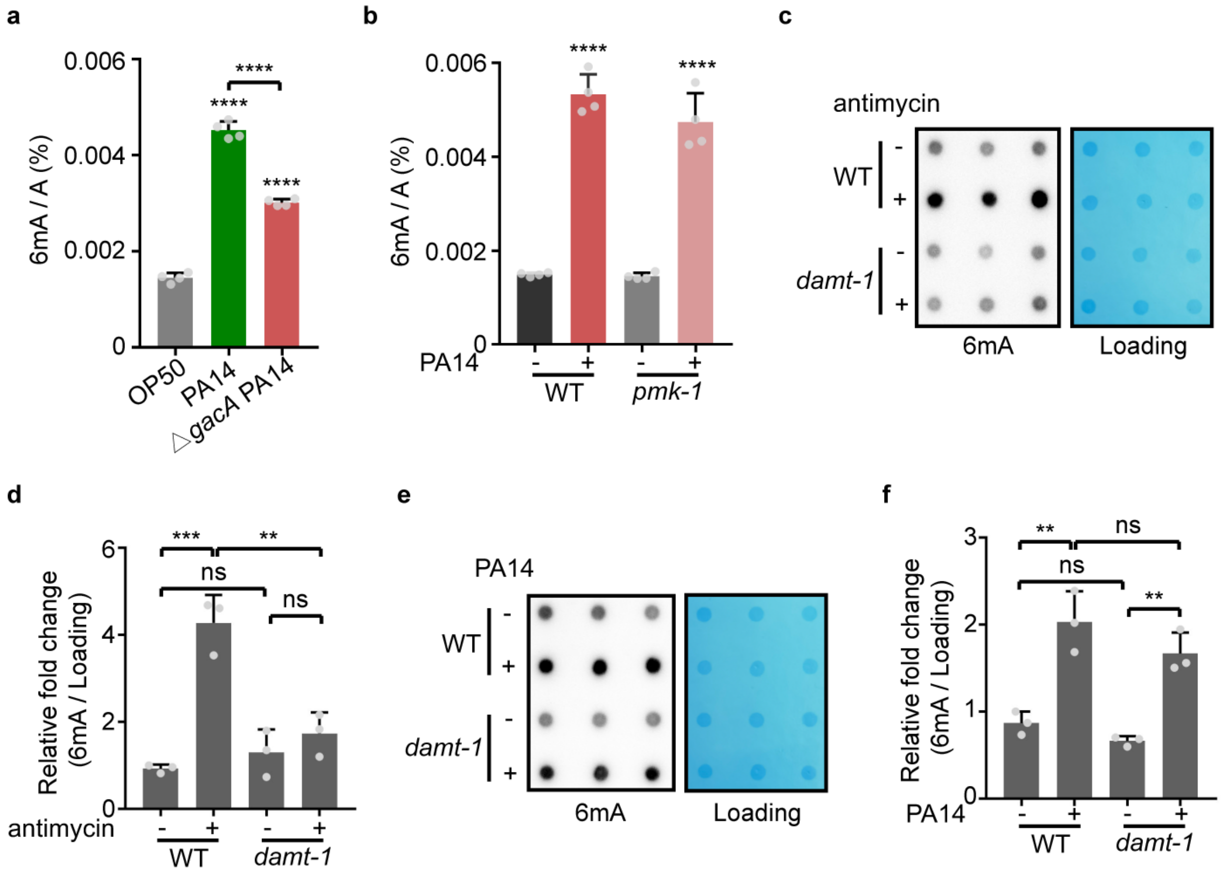

Fig. S1 Levels of 6mA in *C. elegans* genomic DNA are elevated upon infection. a LC-MS/MS analysis of genomic 6mA levels in WT worms fed with OP50, PA14 or *⊿gacA* PA14. $n = 4$. Error bars indicate means + SD. Two-tailed *t*-test, **** $P < 0.0001$. **b** LC-MS/MS analysis of genomic 6mA levels in WT or *pmk-1* mutant worms fed with OP50 or PA14. $n = 4$. Error bars indicate means + SD. Two-tailed *t*-test, ****$P < 0.0001$. **c, d** 6mA dot blotting (**c**) and quantification[66] of WT and *damt-1* animals in the presence or absence of antimycin treatment. $n = 3$. Error bars indicate means + SD. Two-tailed *t*-test, **$P < 0.01$, ***$P < 0.001$. **e, f** 6mA dot blotting (**e**) and quantification (**f**) of WT and *damt-1* animals fed on OP50 or PA14. $n = 3$. Error bars indicate means + SD. Two-tailed *t*-test, **$P < 0.01$.
