# Supplementary material for: A novel N6-Deoxyadenine methyltransferase METL-9 modulates C. elegans immunity via dichotomous mechanisms
Source: Cell Res. 2023 Jun 5;33(8):628–39. doi: 10.1038/s41422-023-00826-y (PMC10397248; doi:10.1038/s41422-023-00826-y)
Supplement: Supplementary file 2 — Supplementary information, Fig. S2 [file 41422_2023_826_MOESM2_ESM.pdf]

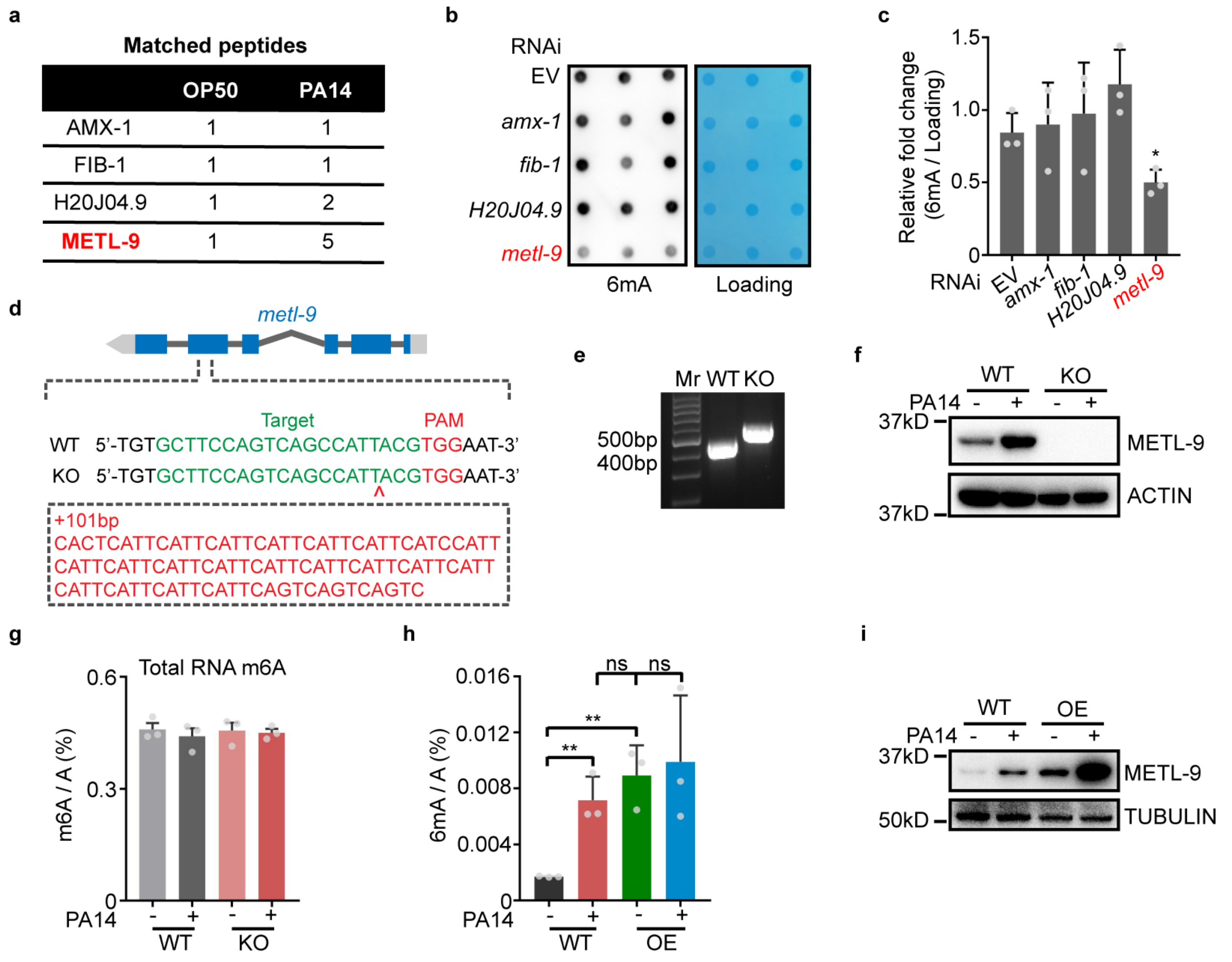

Fig. S2 METL-9 is responsible for 6mA elevation upon infection. a Candidates predicted to be methyltransferases in mass spectrometry analysis. **b, c** 6mA dot blotting (**b**) and quantification (**c**) of genomic DNA from WT animals fed with *amx-1*, *fib-1*, *H20J04.9* or *metl-9* RNAi. Worms fed with bacteria containing the "empty" RNAi vector L4440 (EV) were used as control. $n = 3$. Error bars indicate means + SD. Two-tailed *t*-test, *$P < 0.05$. **d** Generation of the *metl-9* KO strain using the CRISPR/Cas9 approach. A 101 bp insertion in exon 5 of *metl-9* results in a frameshift and a truncated METL-9 protein with 208 amino acids. **e, f** Genotyping (**e**) and western blotting (**f**) of WT and *metl-9* KO strains. **g** LC-MS/MS analysis of total RNA m6A level in WT and *metl-9* KO animals fed on OP50 or PA14. $n = 3$. Error bars indicate means + SD. **h** LC-MS/MS analysis of genomic 6mA levels in WT or FLAG-METL-9 overexpression (OE) worms fed with OP50 or PA14. $n = 3$. Error bars indicate means + SD. Two-tailed *t*-test, **$P < 0.01$. **i** Western blotting of WT and FLAG-METL-9 overexpression (OE) strains fed on OP50 or PA14.
