# Supplementary material for: A novel N6-Deoxyadenine methyltransferase METL-9 modulates C. elegans immunity via dichotomous mechanisms
Source: Cell Res. 2023 Jun 5;33(8):628–39. doi: 10.1038/s41422-023-00826-y (PMC10397248; doi:10.1038/s41422-023-00826-y)
Supplement: Supplementary file 3 — Supplementary information, Fig. S3 [file 41422_2023_826_MOESM3_ESM.pdf]

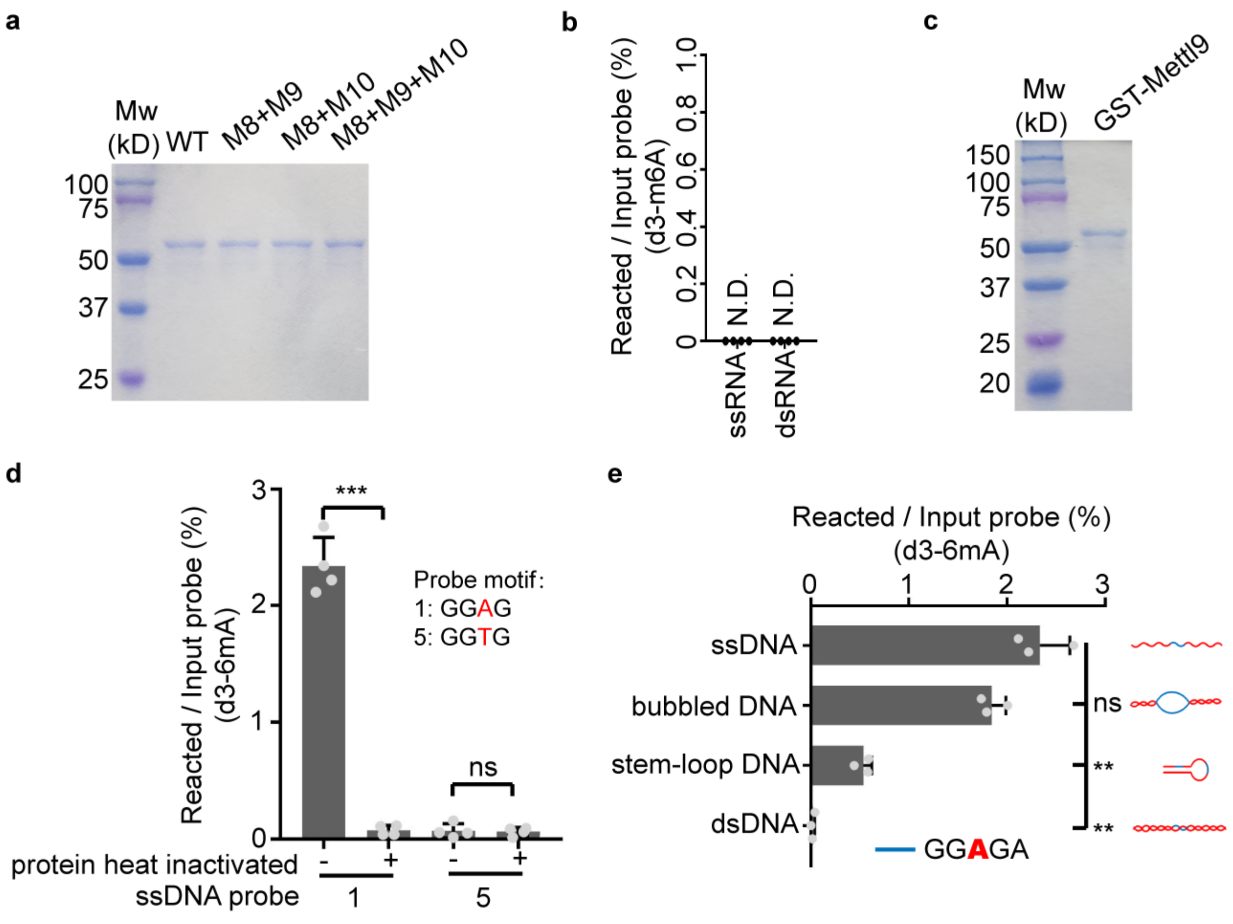

Fig. S3 ***C. elegans*** **METL-9 and mouse Mettl9 display 6mA methyltransferase activity in vitro.** **a** Coomassie blue staining of purified WT and mutant METL-9 recombinant proteins. **b** In vitro RNA m6A methyltransferase activity assay of METL-9 using d3-SAM. **c** Coomassie blue staining of purified mouse Mettl9 recombinant proteins. **d** In vitro DNA 6mA methyltransferase activity assay of mouse Mettl9 recombinant proteins with deuterated SAM (d3-SAM) and ssDNA substrate. Probe 1 and probe 5 are the same probes as in Fig. 5b. $n = 4$. Error bars indicate means + SD. Two-tailed t-test, $***P < 0.001$. **e** DNA substrate preference of mouse Mettl9 analyzed by the in vitro methylation assay using d3-SAM. $n = 3$. Error bars indicate means + SD. Two-tailed t-test, $** P < 0.01$.
