# Supplementary material for: A novel N6-Deoxyadenine methyltransferase METL-9 modulates C. elegans immunity via dichotomous mechanisms
Source: Cell Res. 2023 Jun 5;33(8):628–39. doi: 10.1038/s41422-023-00826-y (PMC10397248; doi:10.1038/s41422-023-00826-y)
Supplement: Supplementary file 4 — Supplementary information, Fig. S4 [file 41422_2023_826_MOESM4_ESM.pdf]

a

Residue site	WT	Mut	Symbol
115	G	E	M1
116	A	D	M2
117	G	D	M3
136	E	K	M4
137	L	R	M5
138	S	P	M6
141	M	R	M7
172	N	K	M8
173	L	D	M9
274	D	G	M10
275	A	E	M11
276	I	R	M12

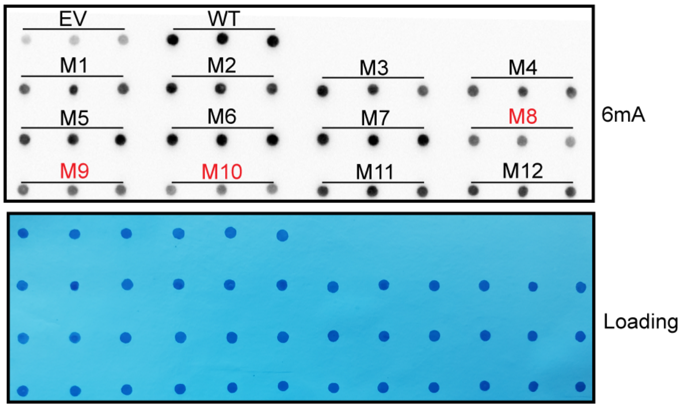

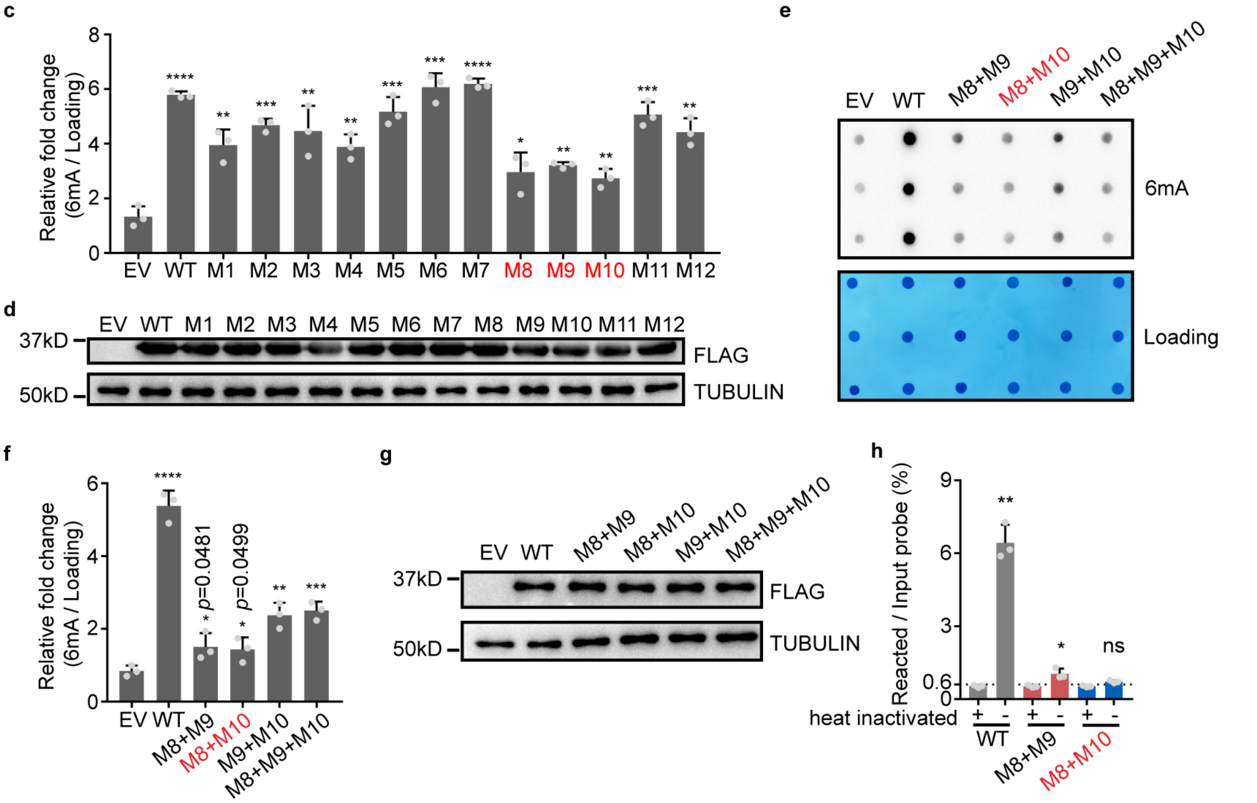

Fig. S4 N172 and D274 residues are essential for the methyltransferase activity of METL-9. **a** Table showing the bioinformatically predicted residues and their mutations. **b–g** 6mA dot blotting (**b, e**), dot blotting quantification of genomic DNA 6mA (*n* = 3) (**c, f**) and anti-FLAG western blotting of whole-cell lysate (**d, g**) from 293T cells with overexpression of WT, mutant FLAG-METL-9 or empty vector (EV). Error bars indicate means + SD. Two-tailed *t*-test, $*P < 0.05$, $**P < 0.01$, $***P < 0.001$, $****P < 0.0001$. **h** LC-MS/MS analysis of genomic 6mA levels in HEK293T cells overexpressing WT or the indicated mutant FLAG-METL-9. $n = 3$. Error bars indicate means + SD. Two-tailed *t*-test, $*P < 0.05$, $**P < 0.01$.
