# Supplementary material for: A novel N6-Deoxyadenine methyltransferase METL-9 modulates C. elegans immunity via dichotomous mechanisms
Source: Cell Res. 2023 Jun 5;33(8):628–39. doi: 10.1038/s41422-023-00826-y (PMC10397248; doi:10.1038/s41422-023-00826-y)
Supplement: Supplementary file 5 — Supplementary information, Fig. S5 [file 41422_2023_826_MOESM5_ESM.pdf]

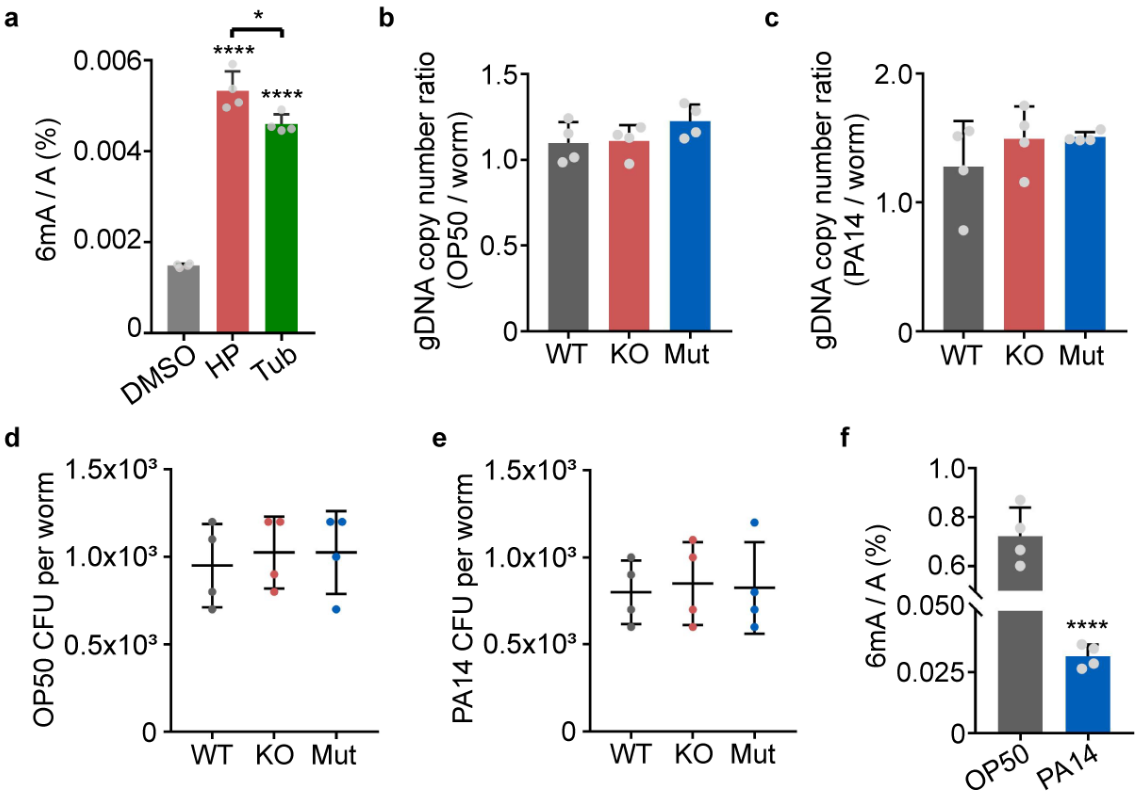

Fig. S5 6mA elevation upon infection is not due to bacterial DNA contamination. a LC-MS/MS analysis of genomic 6mA levels in WT animals treated with hemipyocyanine (HP), tubermycin B (Tub) or DMSO. $n = 4$. Error bars indicate means + SD. Two-tailed t-test, *$p < 0.05$, ****$p < 0.0001$. **b, c** Bacterial genomic DNA contamination assay of WT, *metl-9* KO and *metl-9*^{mut} animals fed on OP50 (**b**) or PA14 (**c**). **d, e** Intestinal CFU assay of WT, *metl-9* KO and *metl-9*^{mut} animals fed on OP50 (**d**) or PA14 (**e**). **f** LC-MS/MS analysis of the 6mA level in OP50 and PA14 genomic DNA. $n = 4$. Error bars indicate means + SD. Two-tailed t-test, ****$p < 0.0001$.
