# Supplementary material for: A novel N6-Deoxyadenine methyltransferase METL-9 modulates C. elegans immunity via dichotomous mechanisms
Source: Cell Res. 2023 Jun 5;33(8):628–39. doi: 10.1038/s41422-023-00826-y (PMC10397248; doi:10.1038/s41422-023-00826-y)
Supplement: Supplementary file 6 — Supplementary information, Fig. S6 [file 41422_2023_826_MOESM6_ESM.pdf]

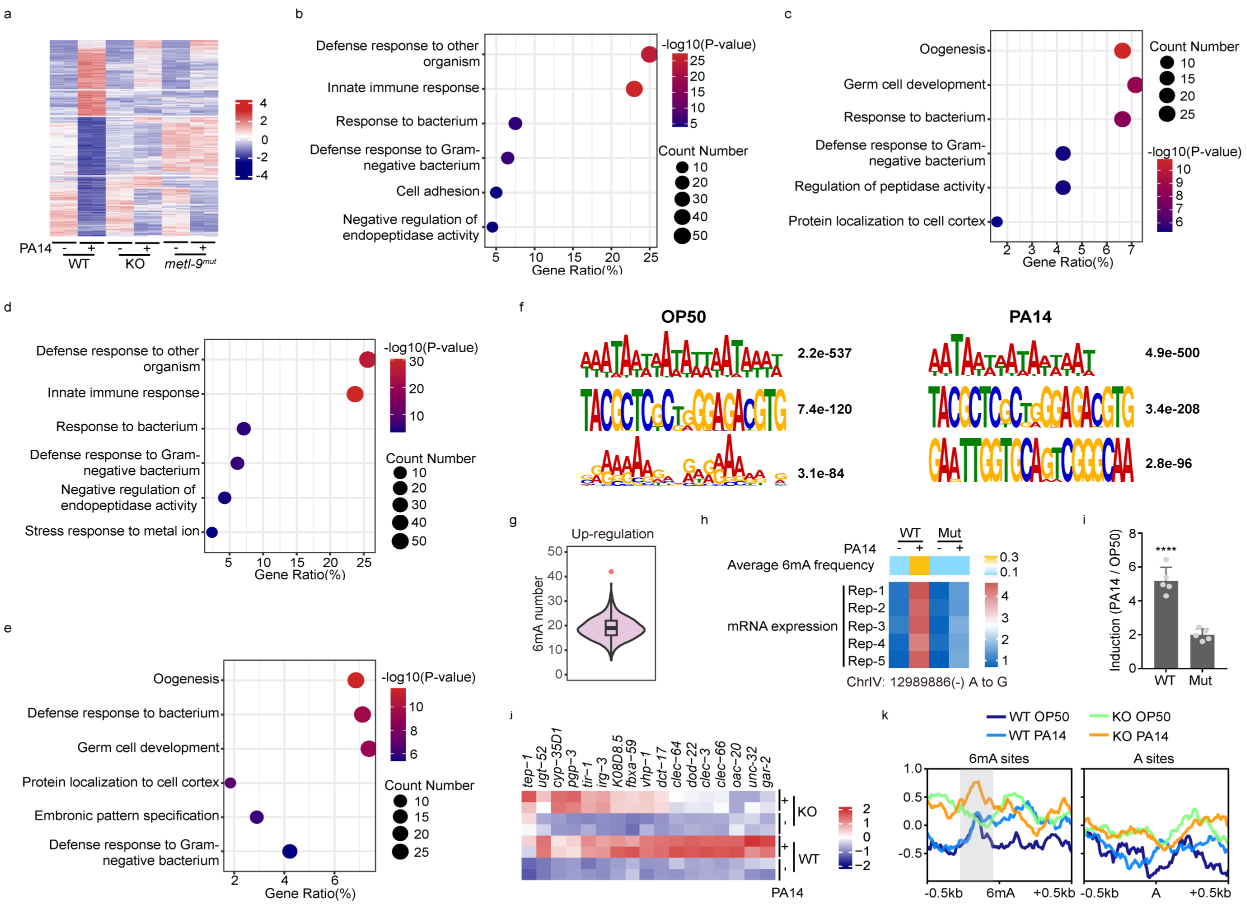

Fig. S6 6mA is a transcriptional modulator of the immune response. a Heatmap of differentially expressed genes in WT, *metl-9* KO and *metl-9*^{mut} animals in the presence or absence of PA14 infection. n = 2. **b, c** Gene Ontology analysis of *metl-9*-dependent upregulated (**b**) or downregulated (**c**) genes. **d, e** Gene Ontology analysis of methyltransferase activity-dependent upregulated (**d**) or downregulated (**e**) genes. **f** METL-9 binding motifs in the absence or presence of PA14 infection. **g** The distribution of the numbers of 6mA sites on expression-unchanged genes after PA14 infection is shown, with the number of 6mA sites located on upregulated genes (FC > 1.5) after PA14 infection highlighted in red. *Monte Carlo* test, P value < 0.001. **h** Heatmap showing average 6mA frequency in the *K10D11.6* promoter region (top) and *K10D11.6* mRNA levels in WT or 6mA site-mutated strains (bottom) in the absence or presence of PA14 infection. **i** The fold induction of *K10D11.6* mRNA levels in WT or 6mA site-mutated strains in the absence or presence of PA14 infection. n = 5. Two-tailed t-test, ****P < 0.0001. **j** Heatmap of upregulated innate immune response genes (FC > 1) with enriched 6mA in WT or *metl-9* KO animals in the presence or absence of PA14 infection. n = 2. **k** H3K27me3 signals around 6mA sites or A sites in WT or *metl-9* KO animals in the absence or presence of PA14 infection.
