# Supplementary material for: A novel N6-Deoxyadenine methyltransferase METL-9 modulates C. elegans immunity via dichotomous mechanisms
Source: Cell Res. 2023 Jun 5;33(8):628–39. doi: 10.1038/s41422-023-00826-y (PMC10397248; doi:10.1038/s41422-023-00826-y)
Supplement: Supplementary file 10 — Supplementary information, Table S4 [file 41422_2023_826_MOESM10_ESM.pdf]

Supplementary information Table S4. Sequences of primers used in this study

Name	Sequence (5'-3')	Annotation
metl-9 RNAi cloning F	TTTCTAGAATGTCATTTCAACTGGAGCGG	
metl-9 RNAi cloning R	TTGCTAGCCTAAAGATCGGTATGTGAAGCATG	
metl-9 qPCR-1F	CAGCCAGAGACAAGTCTCTCAGATG	
metl-9 qPCR-1R	CGATTGCGAGAAACTTGGCGAAC	
metl-9 qPCR-2F	CCTCCTCGATCGCCATTACTCG	
metl-9 qPCR-2R	GCAATCTTGTCCATCTGAGCACC	
H20J04.9 RNAi cloning F	TTTCTAGACTGTTCGCCAACTCATTTAAGCTG	
H20J04.9 RNAi cloning R	TTGCTAGCCCAGTCGTTCTTTCTTCATATCC	
H20J04.9 qPCR-1F	GAGGAGAATCAGTGGGATCCAATGAG	
H20J04.9 qPCR-1R	CATTCATCATCGCTTCGGCCAG	
H20J04.9 qPCR-2F	GGAATGATTGTGAGCAAGTATGGTGC	
H20J04.9 qPCR-2R	GGATCTTTCAACACCGAATACCATTCC	
amx-1 qPCR-1F	CTGAAGCATGTTGTCCATCGTGCT	
amx-1 qPCR-1R	GTTGAGCAGAGAGAGAGTCGGAGG	
amx-1 qPCR-2F	GGTTTCCGATTGCAAGACTGATCGA	
amx-1 qPCR-2R	ACGACATACCGACGAATCGATCAGC	
fib-1 qPCR-1F	GGTGGTGATCGAGGTGGTTTTGG	
fib-1 qPCR-1R	CAACCATGTTCTTTGTGGCAAGAGC	
fib-1 qPCR-2F	CATGCTTGTCGGAATGGTCGATGT	
fib-1 qPCR-2R	CCTCCTTCAGCTTGTTGACTTCACC	
metl-9 WT, KO, mut genotyping F	GCTCAGCTGGAAGGGATACAATG	KO worm contains a 101bp insertion. Mut contains C516G and A922G mutations.
metl-9 WT, KO, mut genotyping R	CCTGTGTGATTGTGGAAGAAGGC	
rpl-32 qPCR-F	AGGGAATTGATAACCGTGTCCGCA	
rpl-32 qPCR-R	TGTAGGACTGCATGAGGAGCATGT	
ssDNA substrate 1	CGTGCTTGCTACTGGTGGGGAGAATGCATGCTACTGGTGC-Biotin	
ssDNA substrate 2	CGTGCTTGCTACTGGTGGGGAGGATGCATGCTACTGGTGC-Biotin	
ssDNA substrate 3	CGTGCTTGCTACTGGTGGGGAGCATGCATGCTACTGGTGC-Biotin	
ssDNA substrate 4	CGTGCTTGCTACTGGTGGGGAGTATGCATGCTACTGGTGC-Biotin	
ssDNA substrate 5	CGTGCTTGCTACTGGTGGGGTGAATGCATGCTACTGGTGC-Biotin	
bubbled DNA F	CGTGCTTGCTACTGGTGGGGAGAATGCATGCTACTGGTGC-Biotin	
bubbled DNA R	GCACCAGTAGCAAGCAAAGAGGGGACCAGTAGCAAGCACG	
dsDNA F	CGTGCTTGCTACTGGTGGGGAGAATGCATGCTACTGGTGC-Biotin	
dsDNA R	GCACCAGTAGCAAGCATTCTCCCCACCAGTAGCAAGCACG	
stem-loop DNA	CGTGCTTGCTACTGGTGGGGAGAATGCATGCTACTGGTGC-Biotin	
ssRNA	CGUGCUUGCUACUGGUGGGGAGAAUGCUUGCUACUGGUGC-Biotin	
dsRNA F	CGUGCUUGCUACUGGUGGGGAGAAUGCUUGCUACUGGUGC-Biotin	
dsRNA R	GCACCAGUAGCAAGCAUUCUCCCCACCAGUAGCAAGCACG	
OP50 specific cloning F	ATGCTAGCCCACATCGGTGTCTGTTATTAACCACA	
OP50 specific cloning R	TGGGTACCAGAACTGCTCTGGATGCATCTCTGGTC	
PA14 specific cloning F	ATGCTAGCGGATGCATTGCACATCTCGCTGTC	
PA14 specific cloning R	TGGGTACCCCTGAAAGTGGTGTGTGGAGATCG	
rpl-32 cloning F	ATGCTAGCAGGGAATTGATAACCGTGTCCGCA	
rpl-32 cloning R	TGGGTACCTGTAGGACTGCATGAGGAGCATGT	
OP50 specific qPCR F	CCACATCGGTGTCTGTTATTAACCACA	
OP50 specific qPCR R	AGAACTGCTCTGGATGCATCTCTGGTC	
PA14 specific qPCR F	GGATGCATTGCACATCTCGCTGTC	
PA14 specific qPCR R	CCTGAAAGTGGTGTGTGGAGATCG	
metl-9 mutation sgRNA1	CCGCTTTCAACCTCCTCGATCGG	
metl-9 mutation sgRNA2	CCAGAATCGCCTTCTTCCACAAT	
metl-9 repair donor sequence	**AAG**CTTCTCGATCGgtaagtgaacagaaactcagattattcaatgaaagctgcaaatttcagCCA<br>TTACTCGCCAGGAAAGCTGTTGAACGACTTGTGGAACGTCGCTCGCC<br>GCTCAAACTGTAATGTCATCGTGTCGCTTGTGCTTCCAGTCAGCCATT<br>ACGTGGAATTCAATCCAAATGGACGAAGCACCCGACCTGACAACTAC<br>CTGAACGTTGCTGGTCGTACCTACGCCGACCATGTTCATCATATGATC<br>GTCAATGTTTTCAAACCGGCAAATTTTGAGGTGCTCAGATGGACAAG<br>ATTGCCGTATCTTTGCGAAGGAGATATGAACAATgtgggtggaagtttgatttga<br>aaattttttcaaacatcaaaatatttcagTCTGCTTACTACCTTCCT**GGC**GCGATCTTTC<br>TTCTGAAACCCATCGAGCCAGAAAGCCCG	**Red**: mutation points
metl-9 KO sgRNA1	GAGAAACCGGCTCAGCTGGA	
metl-9 KO sgRNA3	GCTTCCAGTCAGCCATTACG	
metl-9 KO sgRNA4	GACAACTACCTGAACGTTGC	
K10D11.6 qPCR F	GGTTCAGGACTATTACAACGTCCA	
K10D11.6 qPCR R	TCATTGCTGAAACCGGACCA	
