# Supplementary material for: A novel N6-Deoxyadenine methyltransferase METL-9 modulates C. elegans immunity via dichotomous mechanisms
Source: Cell Res. 2023 Jun 5;33(8):628–39. doi: 10.1038/s41422-023-00826-y (PMC10397248; doi:10.1038/s41422-023-00826-y)
Supplement: Supplementary file 11 — Supplementary information, Table S5 [file 41422_2023_826_MOESM11_ESM.pdf]

Supplementary information Table S5. Worm strains used in this study.

Name	Strain	Source
Bristol N2	N/A	our lab
metl-9 KO	N/A	our lab
metl-9^{mut}	*syb2872*	customized from SunyBiotech
damt-1	*gk961032*	our lab
pmk-1	*km25*	our lab
